# Ryanodine receptor 1-mediated Ca^2+^ signaling and mitochondrial reprogramming modulate uterine serous cancer malignant phenotypes

**DOI:** 10.1186/s13046-022-02419-w

**Published:** 2022-08-11

**Authors:** Li Zhang, Chi-Lam Au-Yeung, Chunxian Huang, Tsz-Lun Yeung, Sammy Ferri-Borgogno, Barrett C. Lawson, Suet-Ying Kwan, Zheng Yin, Stephen T. Wong, Vienna Thomas, Karen H. Lu, Kay-Pong Yip, James S. K. Sham, Samuel C. Mok

**Affiliations:** 1 Department of Gynecologic Oncology and Reproductive Medicine, The University of Texas MD Anderson Cancer Center, Houston, TX 77030 USA; 2 The University of Texas MD Anderson Cancer Center UTHealth Graduate School of Biomedical Sciences at Houston, Houston, TX 77030 USA; 3 Department of Gynecological Oncology, Sun Yat-Sen Memorial Hospital, Sun Yat-Sen University, Guangzhou, China; 4 Department of Pathology, The University of Texas MD Anderson Cancer Center, Houston, TX 77030 USA; 5 Department of Systems Medicine and Bioengineering, Houston Methodist Cancer Center, Weill Cornell Medicine, Houston, TX 77030 USA; 6 Department of Molecular Pharmacology and Physiology, University of South Florida, Tampa, FL 33620 USA; 7 Department of Medicine, Divison of Pulmonary and Critical Care Medicine, Johns Hopkins University School of Medicine, Baltimore, MD 21224 USA

**Keywords:** RYR1, USC, AKT/CREB/PGC-1α signaling pathway and AKT/HK1/2 signaling pathway

## Abstract

**Background:**

Uterine serous cancer (USC) is the most common non-endometrioid subtype of uterine cancer, and is also the most aggressive. Most patients will die of progressively chemotherapy-resistant disease, and the development of new therapies that can target USC remains a major unmet clinical need. This study sought to determine the molecular mechanism by which a novel unfavorable prognostic biomarker ryanodine receptor 1 (RYR1) identified in advanced USC confers their malignant phenotypes, and demonstrated the efficacy of targeting RYR1 by repositioned FDA-approved compounds in USC treatment.

**Methods:**

TCGA USC dataset was analyzed to identify top genes that are associated with patient survival or disease stage, and can be targeted by FDA-approved compounds. The top gene RYR1 was selected and the functional role of RYR1 in USC progression was determined by silencing and over-expressing RYR1 in USC cells in vitro and in vivo. The molecular mechanism and signaling networks associated with the functional role of RYR1 in USC progression were determined by reverse phase protein arrays (RPPA), Western blot, and transcriptomic profiling analyses. The efficacy of the repositioned compound dantrolene on USC progression was determined using both in vitro and in vivo models.

**Results:**

High expression level of RYR1 in the tumors is associated with advanced stage of the disease. Inhibition of RYR1 suppressed proliferation, migration and enhanced apoptosis through Ca^2+^-dependent activation of AKT/CREB/PGC-1α and AKT/HK1/2 signaling pathways, which modulate mitochondrial bioenergetics properties, including oxidative phosphorylation, ATP production, mitochondrial membrane potential, ROS production and TCA metabolites, and glycolytic activities in USC cells. Repositioned compound dantrolene suppressed USC progression and survival in mouse models.

**Conclusions:**

These findings provided insight into the mechanism by which RYR1 modulates the malignant phenotypes of USC and could aid in the development of dantrolene as a repurposed therapeutic agent for the treatment of USC to improve patient survival.

**Supplementary Information:**

The online version contains supplementary material available at 10.1186/s13046-022-02419-w.

## Statement of significance

Our data provides the first evidence that RYR1 upregulation is associated with advanced disease stages in USC patients. The high RYR1 expression plays essential roles during USC progression. We propose inhibition of RYR1 by dantrolene as a novel therapeutic strategy for the treatment of USC patients.

## Background

Uterine serous cancer (USC) is the most common non-endometrioid subtype of uterine cancer. It is also the most aggressive uterine cancer, accounting for no more than 10% of all endometrial cancers but 40% of correlative deaths [1, 2]. In the United States, approximately 5000 new cases are diagnosed and 4000 patients die of the disease annually [3].

USC shares many similarities with high-grade serous ovarian cancer (HGSC), which accounts for most of the 20,000 yearly new cases of epithelial ovarian cancer in the United States [4] including similar age/geographic distribution, risk factors, histopathological features and common genomic signatures such as *TP53* mutation rates [5]. USC is usually sensitive to initial platinum- and taxane-based chemotherapy (> 75% response rates) [6, 7]. However, most of these tumors (> 75–80%) will recur within 12 to 24 months after diagnosis, and these patients will die of progressively chemotherapy-resistant disease [7]. Hence, the development of new therapies that can effectively suppress USC remains a major unmet clinical need.

Drug repositioning, the process of redeveloping a compound for use in different diseases, is a promising field in drug discovery and development [8]. It has many advantages over traditional de novo drug discovery approaches, because repurposed or repositioned drugs have demonstrated safety in humans, therefore, eliminated the need for phase I clinical trials [9] and can significantly reduce the cost and time for drug development. However, repurposed drugs that can be used for USC treatment have not been widely explored.

The purpose of our study was to identify unfavorable prognostic biomarkers associated with disease stage and survival in USC, which can be targeted by FDA-approved repositioned compounds. We demonstrated that over-expression of ryanodine receptor 1 (RYR1), a Ca^2+^ release channel, is associated with advanced disease stages in patients with USC. We further revealed the molecular mechanism by which RYR1 modulates the malignant phenotypes of USC.

Several compounds are known to modulate RYR1 activity, among which dantrolene, a specific blocker of RYR1 and RYR3 [10, 11], was the only one approved by FDA for the treatment of diseases like malignant hyperthermia (MH), and central core disease (CCD) [12]. Dantrolene is extensively applied for reducing muscle pain and stiffness, neuroleptic malignant by blocking ryanodine receptors with minor adverse effects including visual disturbances, headache, insomnia, vomiting, nausea and liver inflammation [12–14]. However, few studies demonstrate the function of dantrolene on tumor. In this study, we demonstrated the feasibility of using dantrolene as a repositioned drug for the treatment of USC.

## Materials and methods

For more details, see Supplemental Experimental Procedures. Reagents and chemicals are summarized in Table S3.

### Cell culture

Human USC cell lines ARK1, ARK2 and HEC50 and human endometrioid endometrial cancer (EEC) cell lines HEC-1A, HEC-1B, HEC-59 and ECC were obtained from American Type Culture Collection (ATCC) and cultured in RPMI-1640 medium (Life Technologies) with 10% fetal bovine serum (FBS) (Life Technologies) and 5 U/mL penicillin and streptomycin (Life Technologies) at 37 °C with a humidified 5% CO_2_ environment. Human uterine epithelial (UE) cells were purchased from LifeLine Cell Technology and cultured in ReproLife™ Reproductive Medium Complete Kit (LifeLine Cell Technology) at 37 °C with a humidified 5% CO_2_ environment.

All cell lines were tested negative for mycoplasma contamination by MycoAlert Mycoplasma Detection Kit (Lonza) and authenticated using the microsatellites panel. Stable RYR1-knockdown and overexpressed ARK1 cells were maintained in RPMI-1640 with 10% FBS, 5 U/mL penicillin and streptomycin and 1 mg/mL puromycin (Invitrogen). All cells were routinely cultured with sub-culturing every 2–3 days.

### Animals and in vivo procedures

All animal procedures described were reviewed and approved by the Institutional Animal Care and Use Committee of The University of Texas MD Anderson Cancer Center. The study is compliant with all relevant ethical regulations regarding animal research. Female nude mice (NCRNU, 6 weeks) were purchased from Taconic Biosciences.

For the drug treatment experiment, luciferase-labeled ARK1 cells were suspended in 100 μL of PBS and intraperitoneally injected into 6-week-old female nude mice. Two weeks later, mice were treated with dantrolene 5 mg/kg or vehicle every other day (*N* = 10/group). After 4 weeks of treatment, in vivo bioluminescence was detected by the IVIS− 200 bioluminescence and fluorescence imaging system (Caliper Life Sciences). At week 6, mice were euthanized, and necropsy was performed. Tumor weight from each mouse was recorded. Tumor tissues were fixed in formalin and processed for histological evaluation. For the knockdown experiment, ARK1 cells stably expressing RYR1 shRNAs or control shRNAs were injected into 6-week-old female mice intraperitoneally. In vivo bioluminescence was monitored every 2 weeks, and signals were recorded. All the mice were euthanized at either week 10 (for tumor progression) or when they were moribund (for survival). For the on-target effect experiment, female athymic nude mice were first injected with luciferase-labeled ARK1 cells stably expressing RYR1 shRNAs (shRYR1) or control shRNAs (shCtrl) intraperitoneally to establish tumor (*N* = 20). Then, half of the mice from each group were treated with dantrolene (5 mg/kg) and the other half with vehicle every other day (*N* = 10). Tumor volume was measured and quantified by in vivo bioluminescence imagingby. After 6 weeks of treatment, mice were euthanized and necropsied.

### siRNAs, shRNAs, CRISPR/Cas9 and virus production

Commercially available siRNAs for human PGC-1α, and corresponding control siRNAs were purchased from Thermo Fisher Scientific. The efficiency PGC-1a silencing was validated by qPCR and Western blotting. Lentiviral transduction particles of two shRNA oligos specially targeting two distinct regions within human RYR1 coding sequence were purchased from Sigma-Aldrich Co. The knockdown efficiency of the two shRNAs were confirmed by qPCR and Western blotting. High-titer CRISPR/dCas9 human RYR1 lentiviral activation particles and control particles were purchased from Santa Cruz Biotechnology, Inc. RYR1 CRISPR/dCas9 activation particles work through a synergistic activation mediator transcription activation system containing a deactivated Ca9 (dCas9) nuclease, activation complex, and target-specific guide RNA. Following transfection, RYR1 expression was examined by qPCR and Western blotting.

### Cell transfection and lentiviral-based gene transduction

For siRNA silencing, ARK1, ARK2 and HEC50 cells were seeded 1 day before 24- to 48-h siRNA (~ 50 μM) transfection using Lipofectamine RNAiMAX reagent (Invitrogen) in Opti-MEM reduced serum medium.

For the generation of stable RYR1-knockdown cell lines, lentiviral particles of shControl (shCtrl), shRYR1–1 and shRYR1–2 were used to infect ARK1, ARK2, and HEC50 at the multiplicity of infection 5 for 24 h. Transduced cells were selected using 3 μg/mL puromycin (Gibco) for approximately 7 days until all control cells died, according to the manufacturer’s protocol. Stable cells derived from single-cell colonies were expanded, frozen, and subjected to indicated experiments.

For the generation of stable RYR1-overexpressing cell lines, ARK1, ARK2, and HEC50 were seeded in a 96-well plate, 4000 cells/well, 24 h prior to virus transduction. Human RYR1 lentiviral particles or control particles were incubated with the cells at the multiplicity of infection 5 for 24 h, followed by puromycin (3 μg/mL) selection for positive clones.

### Cell viability, migration, and apoptosis

Equal numbers of cells were seeded in 96-well plate in triplicate at a density of 4000–5000 cells per well. Cell viability was measured by incubation with MTT reagent (Sigma-Aldrich) at a final concentration of 0.5 mg/mL for 1 h. Absorbance was measured at the wavelength of 570 nm by a microplate reader (BMG LABTECH).

For cell migration, 2000 cells were suspended in serum free RPMI-1640 and seeded in the upper chamber of a 24-well transwell device (Thermo Fisher Scientific). Complete RPMI-1640 with 10% FBS was filled in the lower chamber. After 24–72 h, migrated cells were stained and counted.

For apoptosis, apoptotic cells were detected by annexin V and propidium double staining, followed by flow cytometry using the FITC Annexin V Apoptosis Detection Kit I (BD Pharmingen) according to the manufacturer’s protocol.

### Droplet digital polymerase chain reaction (ddPCR)

Purified RNA was used to synthesize cDNA using High-Capacity cDNA Reverse Transcription Kit (Applied Biosystems). ddPCR analysis was performed using ddPCR Supermix for Probes (no dUTP) (Bio-Rad) on a C1000 Touch Thermal Cycler (Bio-Rad). Droplet was generated by Automated Droplet Generator for Droplet Digital (Bio-Rad). The result was read using the QX200 Droplet Reader (Bio-Rad).

### Reverse-phase protein array (RPPA)

RPPA analysis was performed as described previously with some modifications [15]. In brief, cell pellets were collected and washed. Total proteins were extracted by RPPA lysis buffer. Protein lysis was printed on nitrocellulose-coated slides using Aushon Biosystems 2470 arrayer. 300–500 validated primary antibodies were probed and examined by biotinylated secondary antibodies. Signals were amplified by a Cytomation-catalyzed system of avidin-biotinylated peroxidase (Vectastain Elite ABC kit, Vector Lab). Signals were then visualized by a streptavidin-conjugated HRP and DAB colorimetric reaction and analyzed by the Array-Pro Analyzer software 6.3 (MediaCybernetics) and by SuerCurve_1.5.0 via SuperCurvegUI_2.2.1. All relative protein level data points were normalized for protein loading and transformed to linear values, which can be used for further analysis. Quality control (QC) staining were performed for each antibody and quantified as QC score. A QC score above 0.8 indicates good antibody staining.

### Western blotting analysis

Cells were lysed in radioimmunoprecipitation assay (RIPA) buffer supplemented with protease and phosphatase inhibitors. Protein concentration was measured by BCA assay (Life Technologies). Equal amounts of protein were denatured, subjected to SDS-PAGE and immunoblot assay were performed using indicated primary antibodies overnight at 4 °C. Specific antibody binding was detected by peroxidase-conjugated secondary antibodies (Jackson ImmunoResearch Laboratories). Signals were visualized by chemiluminescence detection kit (Bio-Rad). Beta-actin was applied as a loading control. Density analysis of immune-bands was performed by ImageJ software.

### ATP measurement

CellTiter-Glo 2.0 assay (Promega) was performed to examine ATP production in live cells. A standard curve showing the correlation between cell number and luminescent output was first generated, according to the manufacturer’s protocol. Serial dilutions of cells with 0, 2500, 5000, 10,000, 15,000, and 20,000 cells per well were seeded in a 96-well plate. Equal volume of CellTiter-Glo 2.0 reagent was added to each well. After 10 minutes, luminescence was recorded by a microplate reader (BMG LABTECH).

For RYR1 knockdown and overexpression stable cell lines, 8000 cells per well were seeded in an opaque-walled 96-well plate. ATP measurement was taken after 24 h.

For drug treatment, cells were seeded in a 96-well plate 24 h prior to treatment with 50 μM dantrolene or DMSO, and ATP measurement was taken at 0, 24, 48, and 72 h.

For PGC-1α-silenced RYR1-overexpressed cells, ATP levels were measured 48 h after transfection using CellTiter-Glo 2.0 assay.

ATP Colerimetric/Fluorometric Assay Kit (BioVision #K354) was employed to measure ATP level in mouse tumor tissues. In brief, 20 mg homogenized tissues were lysed in 100 μL ATP Assay Buffer, followed by deproteinization using a deproteinizing sample preparation kit (BioVision #K808) based on the manufacturer’s instructions. Reaction mix (50 μL) was incubated with 50 μL of deproteinized samples for 30 min at room temperature, protected from light. Absorbance (570 nm) or fluorescence (535/587 nm) of standards and samples were measured. ATP amount in tumor tissues were calculated after the sample readings were applied to ATP standard curve according to manufacturer’s protocol.

### Intracellular, endoplasmic and mitochondrial Ca^2+^ measurement

The resting cytosolic [Ca^2+^] was quantified by ratiometric measurement of Fura-2 fluorescence using a Sutter Instrument system. The changes of [Ca^2+^]_i_ induced by 4-Chloro-m-cresol (4-CMC) in AKI cells with or without transfected with shRYR1 or RYR1 were determined using a Leica TCS SP5 confocal fluorescence imaging system as previously described [16–18] . In brief, 5 μM fluo-4 AM (Molecular Probes) was loaded into the cells at room temperature for 30 min. Cells were then washed and waited for another 30 min for deesterification before measurements. Confocal fluorescence images were acquired with a Leica × 63 plan-apochromat objective (numerical aperture 1.2, water immersion). Fluo-4 was excited at 488 nm, and its emission was collected with a spectral window of 495–540 nm at 0.5 or 1 Hz and stored digitally. For mitochondrial [Ca^2+^] measurement, cells were transfected with the mitochondrial specific Ca^2+^ biosensor CEPIA2mt (Addgene, λex: 488 nm, λem: 510–540 nm). For endoplasmic-mitochondrial Ca^2+^ transfer experiment, cells were cotransfected with with mitochrondial Ca^2+^ biosensor CEPIA2mt (Addgene, λ_ex_: 488 nm, λ_em_: 510–540 nm) and the ER Ca^2+^ biosensor R-CEPIA1er (Addgene, λ_ex_: 543 nm, λ_em_: 560–600 nm) [19]. [Ca^2+^]_mito_ and [Ca^2+^]_ER_ were monitored simultaneously using with an Olympus IX81 inverted microscope equipped with 3i spinning disk confocal system. Cells were loaded with caged cyclic ADP-ribose (cADPR) using a reversible permeabilization procedure [18, 20], and photolysis of caged cADPR was induced by the 405 nm laser line at the selected region of interest.

### Intracellular and mitochondrial ROS measurement

Cells were treated with 50 μM dantrolene or DMSO for 72 h. Stable cells were seeded 24 h prior to the measurement. Cells were then digested, washed, and stained by H2DCFDA (BioVision) for 30 min at 37 °C in the dark. Intracellular ROS was estimated via mean fluorescence intensity in Ex/Em 495/529 nm using flow cytometry and compared among different sample groups according to the manufacturer’s description. Mitochondrial ROS was measured using Mitochondrial ROS Detection Assay Kit (Cayman). Cells were stained for 1 h at 37 °C in the dark and analyzed by flow cytometry (Ex/Em: 480–515 nm/560–600 nm).

### Oxygen consumption (OCR) and extracellular acidification rate (ECAR) analysis

OCR, ECAR, and linked ATP production rates were measured by Agilent Seahorse XF Real-Time ATP Rate Assay Kit. Briefly, appropriate numbers of cells were seeded (6 replicates) in XFe96 Cell Culture Microplates at 70–80% confluency in complete RPMI-1640 prior to the analysis. After 24 h, cell culture medium was replaced by Seahorse XF RPMI medium, pH 7.4 with 10 mM XF glucose, 1 mM XF pyruvate and 2 mM XF glutamine. Cells were then incubated in a non-CO_2_ incubator at 37 °C for 45–60 min. The cartridges were preincubated with calibration solution at 37 °C in a non-CO_2_ incubator overnight. Seahorse XF96 analyzer was used to monitor OCR and ECAR values after injections of oligomycin and rotenone/antimycin A. OCR and ECAR were normalized to cell number and protein amount and analyzed by WAVE software (Agilent).

### Targeted metabolomics analysis

For cultured cells, a total of 1 × 10^7^ cells in optimal conditions were seeded in complete RPMI-1640 medium in T75 flasks. After 24–48 h, cells were digested, washed, counted, and frozen in liquid nitrogen or immediately subjected to metabolites extraction and targeted metabolomics analysis for glycolysis and TCA metabolites.

### Mitochondrial membrane potential assay

The mitochondrial membrane potential (ΔΨm) was measured by the JC-1 kit based on the manufacturer’s instructions. In general, 2 × 10^6^ cells were seeded in a 35 mm culture dish and treated with dantrolene or DMSO for 72 h. Stable cells were seeded 24 h prior to the assay. Cells were collected and washed with PBS, followed by staining with JC-1 solution for 30 min at 37 °C. The fluorescence intensity was detected with a flow cytometry at 514/529 nm (Green) and 585/590 nm (Red).

### NAD^+^/NADH assay

NAD^+^ and NADH in human whole cell lysates were measured by NAD/NADH-Glo™ kit following the manufacture’s protocol. Briefly, 6000–7000 cells per well were seeded into a 96 well plate 24–72 h before measurement. Cells were lysed in bicarbonate buffer with 1% dodecyltrimethylammonium bromide (DTAB). To quantify NAD^+^, half of the lysate was mixed with 0.4 N HCl to remove NADH and heated at 60 °C for 15 min, followed by adding Trizma base buffer. The other half of cell lysate was heated at 60 °C for 15 min and mixed with HCl/Trizma buffer to remove NAD^+^. Equal volume of NAD/NADH-Glo detection reagent was added to acid or base treated cell lysates and luminescence was recorded using a luminometer.

### Quantification and statistical analysis

SPSS 24 (IBM Corp) and GraphPad Prism 8.0 (GraphPad Software) software programs were used to perform the statistical tests. All of the in vitro experiments were set up at least 3 samples/repeats per experiment/group/condition. Five fields per slide were selected randomly for the quantification of IHC staining. For in vivo experiments, 6–10 mice per group were used for the analysis. Quantitative data were expressed as mean ± SEM of the indicated number of experiments. Statistical differences were tested by the 2-tailed Student t-test and 1-way or 2-way ANOVA. The Mann-Whitney U test was used for the analysis of nonparametric data. Pearson’s correlation test was used to evaluate the correlation coefficient between 2 parameters. A *p*-value < 0.05 was considered statistically significant. Survival studies were performed by using Kaplan-Meier curves, and *p*-values were calculated by the log-rank test. Microarray data were obtained from The Cancer Genome Atlas (TCGA) public repository (https://www.cbioportal.org/) and the GEO online database (https://www.ncbi.nlm.nih.gov/geo/).

## Results

### RYR1 is an unfavorable prognostic marker for USC

**Fig. 1:**
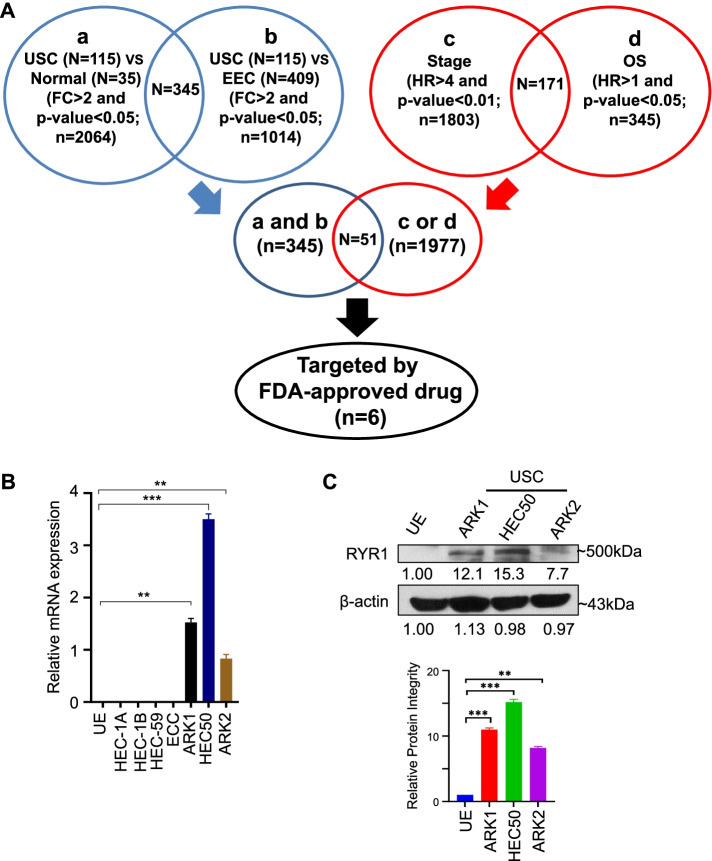
RYR1 is significantly upregulated in USC. **A** Flow chart for identifying differential expressed genes from TCGA dataset that are associated with. USC development including normal control (*n* = 35), EEC (*n* = 409) and USC (*n* = 115). FC=Fold Change, HR = Hazard Ratio, OS=Overall Survival, N = number of sample, n = number of gene. **B** Relative RYR1 mRNA levels in USC, EEC, and UE cells detected by ddPCR. **C** Western blot of RYR1 protein in the indicated USC and UE cells

To exploit the promising therapeutic target candidates associated with advanced disease stages and prognosis in USC, we first identified disease-stage related prognostic markers specific for USC using Significance Analysis of Microarray (SAM) on TCGA dataset (Uterine Corpus Endometrial Carcinoma). 2064 and 1014 genes were upregulated in USC vs Normal control and USC vs EEC comparisons, respectively. In addition, 1977 genes were either significantly correlated with advanced disease stages or overall survival in USC patients by Cox regression analysis. Among these genes, 51 genes overlapped with those that showed significantly higher expression in USC than normal control and EEC, suggesting that they represent prognostic markers specific for USC. In order to identify a repositioned drug for the treatment of USC, we further performed a DrugBank search and found 6 out of 51 genes (RYR1, SCN4A, ANKLE1, KCNQ3, PSAT1 and SLC16A5) can be targeted by FDA-approved drugs (Fig. 1A). We selected RYR1 for further study as it showed the highest fold difference (9.42 folds) between USC and EEC (Table S1).

Next, we asked whether USC cells demonstrated higher RYR1 expression levels than normal counterparts in vitro. ddPCR showed that USC cell lines (ARK1, ARK2, and HEC50) all had significantly higher RYR1 mRNA levels than EEC cell lines (HEC-1A, HEC-1B, HEC-59, and ECC) and normal uterine epithelial (UE) cells (Fig. 1B). Western blot also demonstrated significantly higher RYR1 protein in USC cell lines than UE cells (Fig. 1C).

### RYR1 promotes tumor progression and inhibits apoptosis in USC

**Fig. 2:**
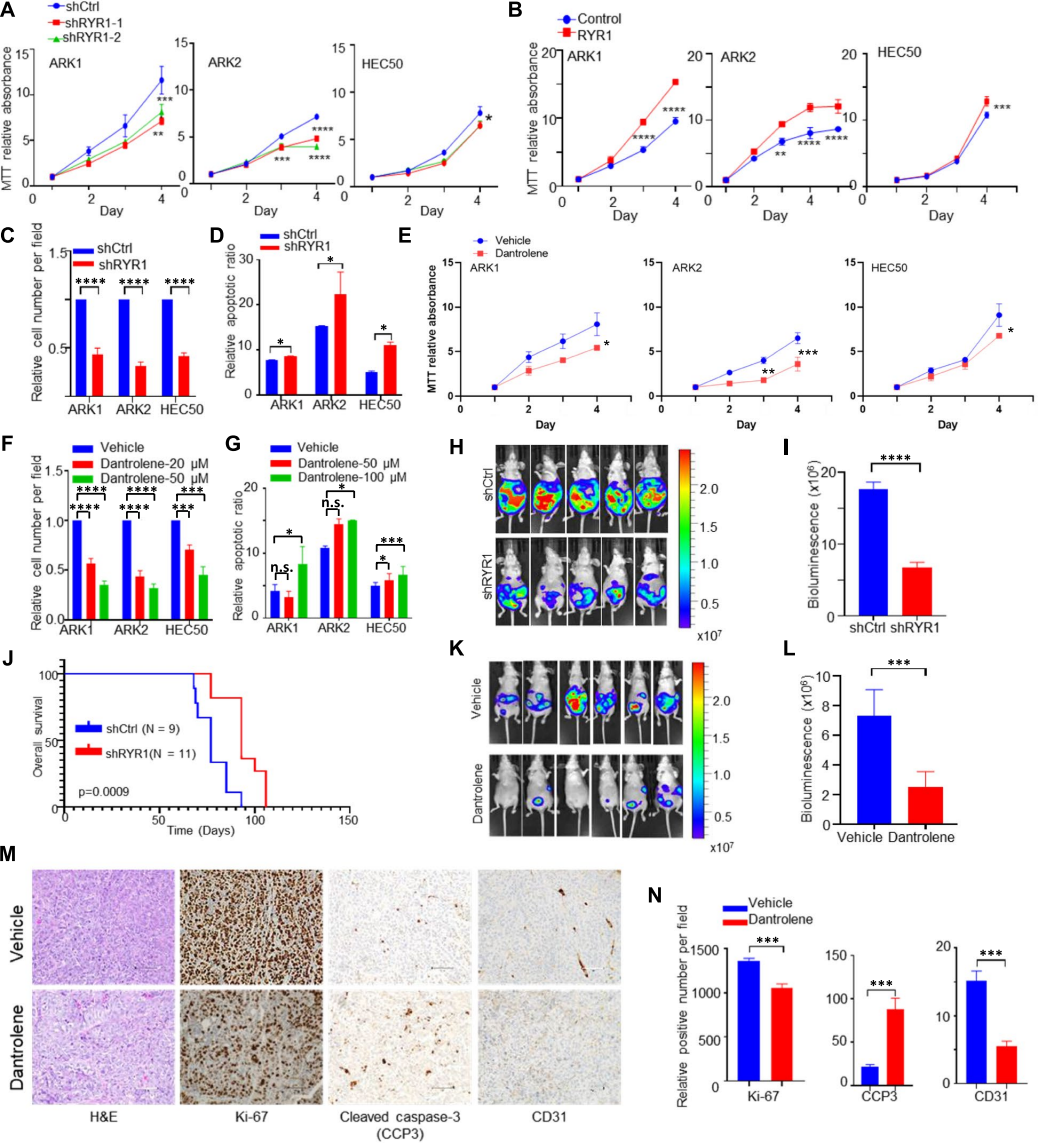
RYR1 enhances cell proliferation and metastasis and suppresses cell apoptosis. **A**, **B** Growth curves showing the effect of (**A**) RYR1 silencing using stably expressed shRNAs (shRYR1–1 and shRYR1–2) and control shRNA (shCtrl) and (**B**) RYR1 overexpression using stably expressed RYR1 construct and empty vector control in USC cells ARK1, ARK2, and HEC50. *p < 0.05, **p < 0.01, ***p < 0.001, ****p < 0.0001 (2-way ANOVA). **C**, **F** Relative number of (**C**) USC shRYR1 or shCtrl cells and (**F**) USC treated with dantrolene (20 or 50 μM) or vehicle migrating through the transwell membrane. **D**, **G** Relative frequencies of apoptotic cells in (**D**) USC shRYR1 or shCtrl cells, and (**G**) USC treated with dantrolene or vehicle. **E** Growth curves showing the effect of dantrolene (30 μM) on USC cells ARK1, ARK2 and HEC50. **H**, **I** Representative bioluminescence images (**H**) and bar charts showing relative bioluminescence intensities (**I**) in nude mice at week 10 after injection with luciferase-labelled ARK1-shRYR1 or shCtrl cells. **J** Kaplan-Meier analysis for overall survival in mice injected with ARK1-shRYR1 or shCtrl. Log rank test. *p* = 0.0009. **K**, **L** Representative bioluminescence images (**K**) and bar charts showing relative bioluminescence intensities (**L**) at week 9 in luciferase-labelled ARK1-bearing mice injected intraperitoneally with dantrolene. *N* = 10 mice per group. M, N. Representative immunohistochemical staining images (**M**) and bar charts (**N**) of numbers of Ki-67-, CD31-, CCP3-positive cells in tumor tissues from mice treated with 50 μM dantrolene or vehicle. Scale bar, 100 μm. In C, D, E, F, G, I, L, N, *p < 0.05, ***p < 0.001, ****p < 0.0001, n.s., no significance (Student t-test). In A-G, N, graphs represent mean ± SEM of 3 independent experiments

*RYR1* encodes the ryanodine receptor type 1 protein predominately found in skeletal muscle cells [21]. It functions as a Ca^2+^ release channel located in the membrane of sarcoplasmic reticulum (SR, muscle cells) or endoplasmic reticulum (ER, non-muscle cells) to mediate the release of Ca^2+^ from the SR/ER. Several drugs or compounds are known to modulate RYR1 activity, such as dantrolene [11], ryanodine [22], ruthenium red [23] and others. Of these, dantrolene is the only specific RYR1 antagonist that is approved by FDA for the treatment of malignant hyperthermia [12]. To determine the role of RYR1 in conferring malignant phenotypes in USC, stable knockdown of RYR1 was performed in USC cell lines ARK1, ARK2 and HEC50 using RYR1-specific small hairpin RNA (shRNA), and RYR1 overexpression was generated by transfecting the full-length RYR1 cDNA construct. Knockdown and overexpression of RYR1 were confirmed by ddPCR and Western blot, respectively (Supplementary Fig. 1A and B). MTT assay demonstrated that RYR1 silencing suppressed cell proliferation in the 3 USC cell lines with higher proliferation rates in RYR1-overexpressing cells (Fig. 2A and B). Depletion of RYR1 caused a reduction in USC cell migration as detected by the transwell assay (Fig. 2C). The apoptotic cell ratio was increased in USC cell lines after RYR1 knockdown as assessed by flow cytometry (Fig. 2D). Conversely, cell migration was enhanced, and apoptosis was suppressed in RYR1 overexpressing cells (Supplementary Fig. 1C and D). In contrast, dantrolene treatment did not significantly decrease cell viability in EEC cell lines HEC1A and HEC1B (Supplementary Fig. 1E), which do not express detectable levels of RYR1 mRNA. These findings indicate RYR1 is a potential common pharmaco-therapeutic target for suppressing the malignant phenotypes of USC cells.

We examined whether dantrolene could potentially be repurposed for the suppression of the USC phenotypes, as observed in RYR1-silenced USC cells. Application of dantrolene exhibited a significant inhibitory effect on the proliferation and mobility of USC cells (Fig. 2E and F). Furthermore, flow cytometry demonstrated that dantrolene-treated USC cells had significantly higher numbers of apoptotic cells than controls (Fig. 2G). To confirm the on-target effect of dantrolene in vitro, RYR1-silenced ARK1 cells were treated with dantrolene (50 μM) or vehicle solution, and cell growth was monitored. Significant inhibitory effect of dantrolene was observed in the mock transfectants instead of RYR1 shRNA transfected ARK1 cells (Supplementary Fig. 1F). These findings imply that RYR1 mediates the effect of dantrolene in suppressing the malignant phenotype in USC cells.

To examine the tumor-promoting effect of RYR1 in vivo, luciferase-labeled RYR1 shRNA or mock transfected ARK1 cells were intraperitoneally injected into 6-week-old female nude mice. Tumor progression was examined by measuring bioluminescence every 2 weeks (Fig. 2H). Mice injected with RYR1-silenced ARK1 cells had significantly lower bioluminescence signals detected at week 10, compared to those injected with control cells (Fig. 2I). Survival analysis showed that mice injected with RYR1-silenced ARK1 cells had significantly longer overall survival times than controls (Fig. 2J), supporting the hypothesis that RYR1 plays a crucial role in USC progression. Similarly, mice were injected intraperitoneally with dantrolene (5 mg/kg) or vehicle 3 times per week for 5 weeks, 28 days after luciferase-labeled ARK1 cell inoculation to check dantrolene effect in vivo. Dantrolene-treated group had significantly lower bioluminescence signals than control group (Fig. 2K and L). There was no significant difference in the body weight of dantrolene- and vehicle-treated mice (Supplementary Fig. 1H), suggesting minimal toxicity of dantrolene in the mice.

To further determine the effect of RYR1 silencing and dantrolene treatment on tumor cell proliferation, angiogenesis, and apoptosis, immunolocalization (IHC) of Ki-67, CD31, and cleaved caspase 3 (CCP3) were performed on tumor tissues harvested in the mouse models. USC tumors of the RYR1 knockdown group and the dantrolene-treated group had significantly lower numbers of Ki-67- and CD31-positive cells and higher numbers of CCP3 cells compared to controls (Fig. 2M and N, and Supplementary Fig. 1I). We also confirm the on-target effect in vivo, female athymic nude mice were first injected with luciferase-labeled ARK1 cells stably expressing RYR1 shRNAs or control shRNAs intraperitoneally to establish tumor. They were then treated with dantrolene or vehicle every other day for 6 weeks. There was a significant decrease in bioluminescence signals in the RYR1-silenced group and dantrolene-treatment group compared to the control group. However, there is no significant difference in tumor growth in RYR1-silenced group with or without dantrolene treatment (Supplementary Fig. 1G), which is similar to what we observed in vitro. Taken together, our results strongly support the tumorigenic role of RYR1 in USC and the feasibility of repurposing dantrolene as an effective agent for the treatment of USC.

### RYR1 modulates intracellular calcium signaling in USC cells

**Fig. 3:**
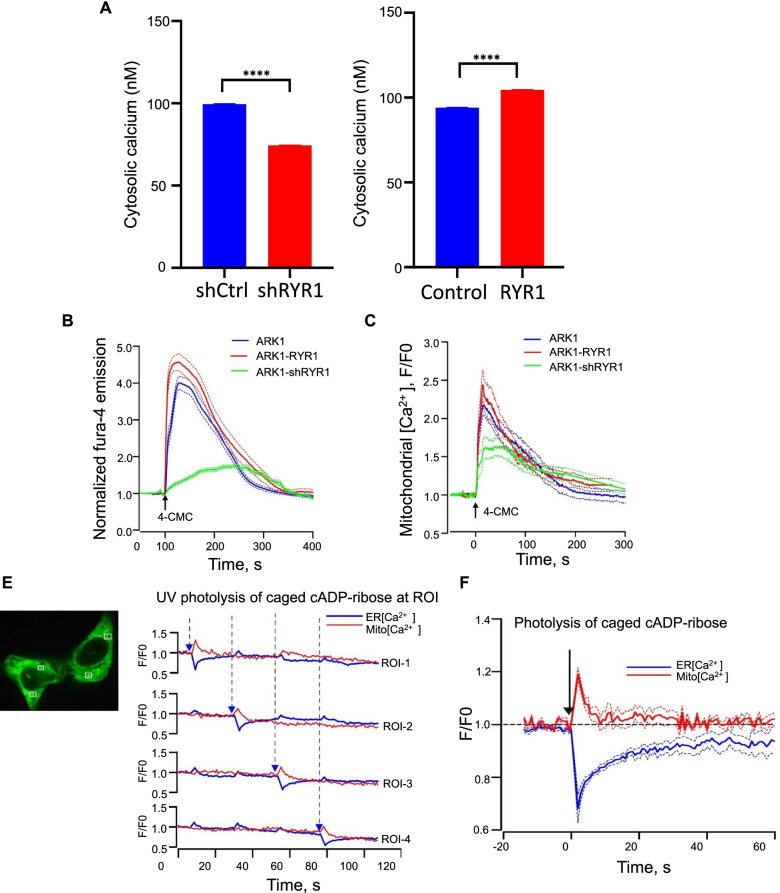
RYR1 modulates intracellular calcium levels. **A** Cytosolic Ca^2+^ concentration (nM) monitored by Fura-2 in ARK1-shRYR1 (*n* = 87) or -shCtrl (*n* = 58) (left) and -RYR1 (*n* = 271) or -Control (*n* = 171) (right) cells. ****p < 0.0001 (Student t-test). **B** Mean normalized RYR1- mediated Ca^2+^ transient activated by 4-CMC (25 μM) in ARK1 (blue, *n* = 116), ARK1-shRYR1 (red, *n* = 70), and ARK1-RYR1 (green, *n* = 55) cells. **C** Mitochondrial Ca^2+^ detected by CEPIA2mt in ARK1 (blue, *n* = 21), ARK1- RYR1 (red, *n* = 16), and ARK1-shRYR1 (green, *n* = 13) cells activated by 4-CMC (25 μM). **D** Representative image of 2 ARK1 cells, indicating 4 different ROIs at which photorelease of cADPR was activated, and ER and mitochondrial Ca^2+^ transient was recorded. The time course of change in ER (blue) and mitochondrial (red) Ca^2+^ signals (F/F0) recorded in the 4 ROIs depicted in the image. **E** Mean normalized ER [Ca^2+^] and mitochondrial [Ca^2+^] activated by photorelease of caged cADPR at subcellular ROIs in ARK1 cells (*n* = 12 ROIs in 9 cells)

To investigate the RYR1-dependent changes in Ca^2+^ homeostasis of USC cells, cytosolic [Ca^2+^] was first determined by radiometric measurement of Fura-2 fluorescence in ARK1-shCtrl, ARK1-shRYR1, ARK1-Control and ARK1-RYR1 cells. The resting [Ca^2+^]_i_ was significantly suppressed in the shRYR1 cells (shCtrl: 99.4 ± 2.1 nM, *n* = 58; shRYR1: 74.4 ± 3 nM, *n* = 87; *p* < 0.001) and significantly elevated in RYR1-overexpressing cells (Control: 94 ± 3.2 nM, *n* = 171; RYR1: 104.5 ± 2.2 nM, *n* = 217; p < 0.001; Fig. 3A), suggesting the differences in resting [Ca^2+^]_i_ in the indicated cells were related to Ca^2+^ signals mediated by RYR1.

The activity of RYRs was further examined by the RYR agonist 4-Chloro-m-cresol (4-CMC). Application of 4-CMC (5 μM) caused a rapid transient increase in cytosolic [Ca^2+^]. The responses were significantly suppressed in the shRYR1 cells and accentuated in the RYR1-overexpressing cells (Fig. 3B). These results suggested that functional RYR1 is expressed and operated as an important determinant of Ca^2+^ homeostasis in ARK1 cells. RYR1 may modulate mitochondrial [Ca^2+^] ([Ca^2+^]_m_) through ER-mitochondrial Ca^2+^ transfer [24]. ER-mitochondrial Ca^2+^ transfer was monitored in ARK1 cells transfected with the mitochondrial Ca^2+^ biosensor CEPIA2mt. Application of 4-CMC caused a rapid increase in [Ca^2+^]_m_ in the control ARK1 cells. The response was enhanced in the RYR1-overexpressing cells and was significantly suppressed in ARK1 cells transfected with shRYR1 (Fig. 3C). In a separate set of experiments, ARK1 cells were transfected with both the ER specific Ca^2+^ biosensors G-CEPIA1er and CEPIA2mt for simultaneous recording of ER [Ca^2+^] ([Ca^2+^]_ER_) and [Ca^2+^]_m_, respectively. Activation of RYRs by photorelease of caged cADP-ribose (cADPR), an endogenous activator of RYRs, at a subcellular region of interest (ROI) caused an immediate transient reduction in [Ca^2+^]_ER_, which was associated with a fast, transient increase in [Ca^2+^]_m_ (5–10 s) followed by a reduction in the mitochondrial Ca^2+^ signal (Fig. 3D and E). The ER-mitochondrial Ca^2+^ transfer occurred locally within the ROI undergoing photolysis, without affecting [Ca^2+^]_ER_ or [Ca^2+^]_m_ in the other ROIs. These experiments clearly demonstrated that RYR is capable to mediate ER-mitochondrial Ca^2+^ transfer in ARK1 cells.

### RYR1 silencing down-regulates mitochondrial genes in USC

To identify the RYR1-mediated signaling network that modulates the malignant phenotypes of USC, unbiased reverse-phase protein array (RPPA) was performed on over 400 proteins of key signaling networks. Cell lysates from RYR1 shRNA and scramble shRNA-transfected ARK1 cells as well as dantrolene- and vehicle-treated ARK1 cells were used to identify the overlapping differentially expressed proteins that are associated with down-regulation of RYR1 expression and activity. A total of 305 downregulated and 292 upregulated proteins were found. Among them, 64 downregulated and 38 upregulated proteins were common in both experimental treatments (Supplementary Fig. 2A).

**Fig. 4:**
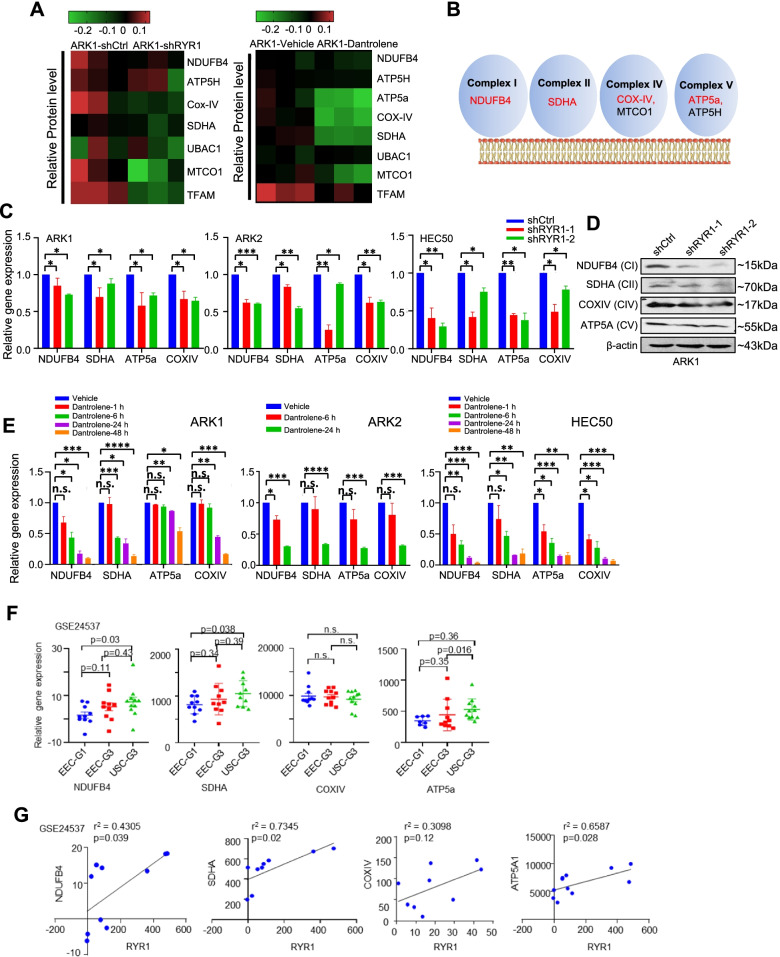
RYR1 depletion suppresses mETC gene expression. **A** Heatmaps showing relative expression levels of mitochondrial proteins measured by RPPA in ARK1-shCtrl and shRYR1 cells (left); or cells treated with vehicle or dantrolene (right). Signal intensities were normalized to the control groups. **B** Graphic illustration of components in the 4 complexes of mitochondrial respiration chain. **C**, **D**. Relative mRNA (**C**) and protein (**D**) expressions of mETC genes determined by qPCR and Western blot, respectively, in USC cells transfected with RYR1-specific shRNAs or shCtrl. β-actin was used as a loading control. Data are shown as the mean ± SEM of 3 independent experiments. **E** Relative mRNA expression of mETC genes determined by qPCR in USC cells treated with 50 μM dantrolene or vehicle. Data are shown as the mean ± SEM of 3 independent experiments. *p < 0.05, **p < 0.01, ***p < 0.001, ****p < 0.0001, n.s., no significance (Student t-test). **F** Relative expressions of NDUFB4, SDHA, COXIV and ATP5a in TCGA dataset (Student t-test). **G** Correlation of NDUFB4, SDHA, COXIV and ATP5a with RYR1 in GSE24537

Our attention was drawn to proteins in the top rank of the overlapping list (Fig. 4A and B). They included components of mitochondrial electron transport chain (mETC) (e.g., COXIV, NDUFB4, ATP5a, SDHA), suggesting the potential role of RYR1 in modulating mitochondrial functions. The RPPA results were validated by qPCR and Western blot demonstrating that the USC cells stably transfected with RYR1 shRNA had significantly lower mRNA and protein levels of NDUFB4 (Complex I), SDHA (Complex II), COXIV (Complex IV) and ATP5a (Complex V) than the mock transfectants (Fig. 4C and D). Furthermore, dantrolene (50 μM) treatment of USC cells caused significant time-dependent reduction in these mETC gene mRNA levels (Fig. 4E).

To further determine the role of RYR1 in regulating mETC gene expression in USC tissue samples, regression analysis was performed using TCGA datasets to determine the correlation between mRNA expression of RYR1 and the mETC genes. The results showed significantly higher expression of NDUFB4, SDHA and ATP5a in USC than in grade 1 and grade 3 EECs (Fig. 4F). Significant positive correlations were also observed between the expression of RYR1 and the mETC genes in USC, except COXIV (Fig. 4G). In addition to the above 4 mETC genes, we found positive correlations between RYR1 and some other members of NDUF, SDH, COX, and mitochondrial ATPase subfamilies in GEO datasets (GSE24537) (Table S2), suggesting that these mETC genes may play a role in mediating the tumor-promoting effect of RYR1 in USC.

### RYR1 modulates mitochondrial bioenergetics properties in USC

**Fig. 5:**
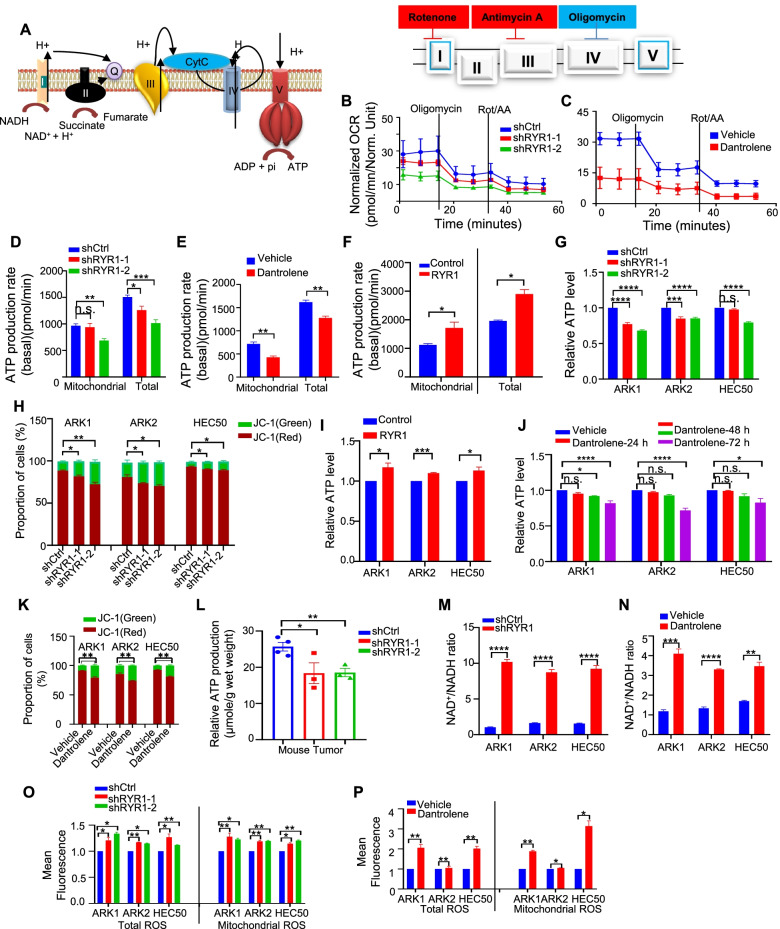
RYR1 modulates mitochondrial bioenergetics properties. **A** Graphic illustration of ETC functions in mitochondrial membrane. **B**-**C** Representative pattern of OCR as a function over time in ARK1-shRYR1 vs shCtrl cells (**B**) or dantrolene- vs vehicle-treated cells (**C**). **D**-**F** Mitochondrial ATP and total ATP production rate (basic, pmol/min) in ARK1- shRYR1 or -shCtrl cells (**D**), cells treated with dantrolene or the vehicle (**E**), and ARK1-RYR1 or -Control cells (**F**). **G**, **I**, **J** Relative ATP levels in ARK1-shRYR1 vs shCtrl cells (**G**), USC RYR1 vs control cells (**I**), indicated cells incubated with dantrolene or vehicle (**J**). **H**, **K** Mitochondrial membrane potential of USC cells transfected with shRYR1 or shCtrl (**H**), treated with 50 μM dantrolene or vehicle (**K**) examined by JC-1 distribution. Green: JC-1 monomer, Red: JC-1 aggregates. **L** Bar charts showing relative ATP production (μmol/g) in mouse tumor tissues derived from ARK1-shRYR1 or -shCtrl cells. M-N, Relative NAD+/NADH ratios in USC cells transfected with shRYR1 or shCtrl (**M**) and treated with dantrolene or the vehicle (**N**). **O**-**P** Relative total and mitochondrial ROS in USC cells transfected with shRYR1 or shCtrl (**O**) and treated with dantrolene or vehicle (**P**) In B-P, *N* = 3 independent experiments. Data represent mean ± SEM. *p < 0.05, **p < 0.01, ***p < 0.001, ****p < 0.0001, n.s., no significance (Student t-test)

Our results demonstrated that RYR1 regulated the expression of key mETC components (Fig. 5A) in USC cells, suggesting that RYR1 may modulate mitochondrial bioenergetics in these cancer cells. To test this hypothesis, the RYR1-dependent regulation of oxidative phosphorylation (OXPHOS) was determined in ARK1 cells. Oxygen consumption rate (OCR) and ATP production rate were first determined by a Seahorse Analyzer. The results showed that there was a significant reduction in OCR (Fig. 5B and C), and basal mitochondrial and whole cell ATP production rates (pmol/min) (Fig. 5D and E) in shRYR1-transfected or 50 μM dantrolene-treated ARK1 cells. In contrast, ATP production rate was significantly higher in RYR1 stably transfected cells than the mock transfectants (Fig. 5F).

Next, we determined total live-cell ATP production and mitochondrial membrane potential (MMP) in USC cells using CellTiter-Glo kit and JC-1 assay. Tetrachloro-teraethyl benzimidazole carbocyanine iodide (JC-1) is a cationic dye that accumulates in mitochondria, which is commonly used for monitoring MMP (**Δ**ψ_m_). Usually, under low MMP, JC-1 exists as monomer and yields green fluorescence. The dye will aggregates under MMP leading to red fluorescence [25]. RYR1 depleted ARK1 cells had significantly lower total live-cell ATP and MMP (Fig. 5G and H) with contrast result in RYR1 overexpression cells (Fig. 5I and Supplementary Fig. 3A). Furthermore, USC cell lines treated with dantrolene (50 μM) for different durations showed that dantrolene decreased live-cell ATP levels in a time-dependent manner (Fig. 5J). A significant decrease in MMP was also observed in dantrolene-treated USC cells (Fig. 5K). ARK1-shRYR1 cells induced mouse tumor tissues also demonstrated a significantly reduced ATP production compared to control (Fig. 5L).

To further examine the role of RYR1 on OXPHOS, the effect of RYR1 expression on 2 additional key OXPHOS-related parameters—NAD^+^/NADH ratio and accumulation of reactive oxygen species (ROS) were determined. The results showed significantly higher NAD^+^/NADH ratio, mitochondrial and total cellular ROS in RYR1-silenced or dantrolene-treated USC cells than control cells (Fig. 5M-P) with contrasting results in RYR1-overexpressing cells (Supplementary Fig. 3B and C). Taken together, these results demonstrate that RYR1 alters mitochondrial biogenetics properties through modulating OXPHOS process in USC.

### RYR1 silencing suppresses glycolysis and TCA cycle in USC

**Fig. 6:**
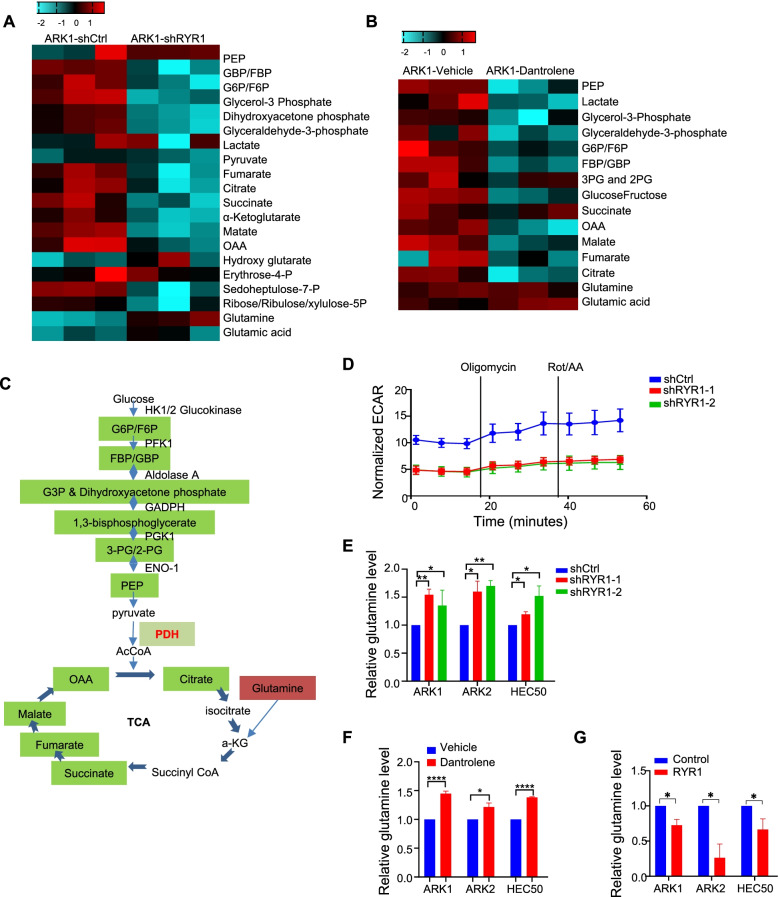
RYR1 silencing suppresses TCA cycle and glycolysis. **A**-**B** Heatmaps showing relative levels (-log2) of differential metabolites in glycolysis and TCA cycle in cell lysates. ARK1-shCtrl vs ARK1-shRYR1 (**A**) and ARK1-vehice vs ARK1-dantrolene (**B**), subjected to LC-MS for metabolite measurement. **C** Graphic illustration of intermediate metabolites involved in glycolysis and TCA cycle. Green- labeled indicate the decreased ones in RYR1-silenced or dantrolene-treated cells than controls. **D** Representative patterns of ECAR from ARK1 cells transfected with shRYR1 or shCtrl. **E**-**F** Relative glutamine levels in USC cells transfected with RYR1- shRNAs or shCtrl (**E**) and treated with 50 μM dantrolene or vehicle (**F**). **G** Relative glutamine levels in USC cells transfected with RYR1 full-length cDNA or control vector. In D-G, data represent the mean ± SEM of 3 independent experiments. *p < 0.05, **p < 0.01, ***p < 0.001, ****p < 0.0001 (Student t-test)

To exploit energy metabolism alterations driven by RYR1, targeted metabolomics analysis was performed using liquid chromatography-tandem mass spectrometry (LC-MS/MS) in RYR1-deficient ARK1 cells and control cells. The results revealed a markedly lower concentration of glycolytic and TCA metabolites, such as G6P/F6P, FBP/GBP, citrate, succinate and malate in both RYR1 knockdown and 50 μM dantrolene-treated cells, suggesting that removal of RYR1 impeded the normal operation of the glycolysis and TCA cycle in ARK1 cells (Fig. 6A-C). Extracellular acidification rate (ECAR), which measures the excretion of lactic acid per unit time in RYR1 knockdown ARK1 cells was determined. ECAR of the shRYR1-transfected ARK1 cells was significantly lower than controls (Fig. 6D).

Interestingly, we found that glutamine levels were significantly increased in RYR1-knockdown and dantrolene-treated ARK1 cells from LC-MS/MS (Fig. 6A and B), suggesting that increased glutamine uptake could be a feedback mechanism by which the cells compensated for the shutting down of both OXPHOS and glycolysis due to RYR1 depletion. In congruent with the LC-MS experiments, glutamine level was significantly elevated after RYR1 silencing or blockage by dantrolene (Fig. 6E and F) and decreased in RYR1-overexpressing cells (Fig. 6G), suggesting that high levels of RYR1 can probably speed up glycolysis and TCA cycle, which does not need glutamine compensation.

### Ca^2+^ dependent AKT/CREB/PGC-1α and AKT/HK1/2 axes is essential for RYR1-mediated USC progression

**Fig. 7:**
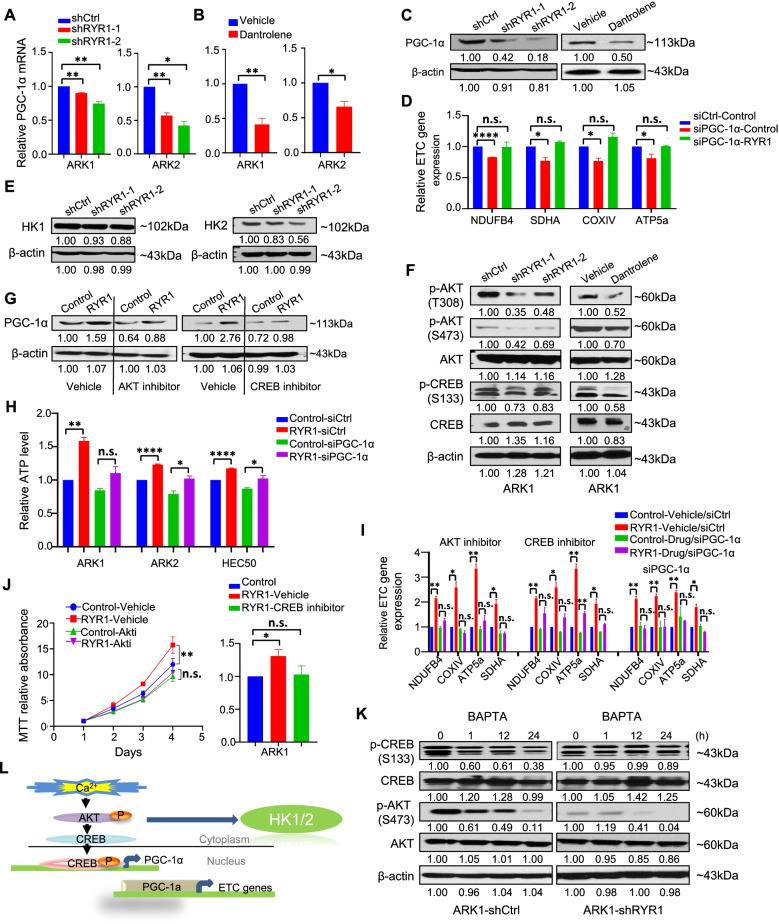
Ca^2+^ /AKT/CREB/PGC-1α axis is essential for tumor progression. **A**-**B** PGC-1α mRNA expression in the indicated cells with shCtrl or shRYR1 (**A**) or incubated with dantrolene or vehicle (**B**). **C** **F** Western blot of PGC-1α (**C**) proteins in AKT/CREB pathway (**F**) in control- or RYR1-silenced and vehicle- or dantrolene-treated ARK1 cells. **D** mETC genes mRNA expression in PGC-1α-silenced (siPGC-1α) or control (siCtrl) ARK1 cells transfected with RYR1 or control. **E** Western blot of HK1 and HK2 in control- or RYR1- silenced ARK1 cells. **G** Western blot of PGC-1α in ARK1-Control and ARK1-RYR1 cells treated with of AKT or CREP inhibitor vs vehicle. **H** Relative ATP level in control and RYR1-overexpressing cells transfected with siCtrl or siPGC-1α. *p < 0.05, **p < 0.01, ****p < 0.0001 (Student t-test). **I** mETC genes mRNA expression in ARK1- Control and ARK1-RYR1 cells in the presence of siPGC-1α, AKT (0.1 μM) or CREB inhibitor (0.1 nM) or controls. **J** Cell growth of ARK1-Control and ARK1-RYR1 incubated with AKT or CREB inhibitor. *p < 0.05, **p < 0.01, n.s., no significance (2-way ANOVA). **K** Western blot of phosphorylated AKT (S473), phosphorylated CREB (S133), and total AKT and CREB in ARK1-shCtrl and ARK1-shRYR1 cells treated with BAPTA or vehicle. **L** Graphic illustration of the molecular mechanism. In A, B, D, I, *N* = 3 independent experiments. HPRT was internal control. *p < 0.05, **p < 0.01, ****p < 0.0001, n.s., no significance (Student t-test). In C, E, F, G, K, *N* = 3 independent experiments. Data are shown as mean ± SEM. ImageJ software was applied to quantify signals. β-actin was used as loading control

Peroxisome proliferator-activated receptor coactivator protein-1alpha (PGC-1α) has been shown to bind directly to the promoter regions of mETC genes to upregulate transcription in breast and ovarian cancer cells [26]. We examined whether PGC-1α mediated the effect of RYR1 in regulating mETC expression. Both PGC-1α mRNA and protein levels were significantly lower in ARK1 and ARK2 cells transfected with RYR1 shRNA or treated with dantrolene (50 μM) (Fig. 7A-C). Furthermore, PGC-1α silencing and rescue experiments were performed by overexpressing RYR1 in PGC-1α–silenced ARK1 cells, and mETC genes expression was determined using qPCR. We found that the decrease of mETC genes in PGC-1α-knockdown cells can be compensated by overexpressing RYR1 in those cells (Fig. 7D). These results clearly suggest that RYR1 may indeed regulate PGC-1α expression to modulate the expression of mETC genes in USC cells.

Next, we determined whether hexokinases (HKs), which are key enzymes that catalyze the first step of glycose metabolism by converting glucose to glucose-6-phosphate (G6P), mediate the effect of RYR1 in regulating glycolysis and TCA cycle. Of the HKs, HK1 and HK2 are the 2 predominant isoforms in most human tissues [27]. Western blot showed markedly lower levels of total HK1 and HK2 in RYR1-silenced ARK1 cells than in control cells (Fig. 7E). To further determine the molecular mechanism by which PGC-1α and HK1/2 are regulated by RYR1, we screened all the known Ca^2+^-dependent signaling molecules and transcription factors from our RPPA and public datasets. We focused on Ca^2+^/cAMP response element (CRE)-binding protein (CREB), which upon activation can form a functionally active dimer that binds to the cis-acting CRE element within the promoters of target genes. The PPARGC1A gene contains a CREB binding domain within its promoter. CREB phosphorylated by AKT is able to directly interact with this domain and trigger PGC-1α transcription [28]. Our RPPA data demonstrated that RYR1 shRNA-transfected and dantrolene-treated ARK1 cells had significantly lower levels of phosphorylated AKT and CREB than controls (Supplementary Fig. 4A). This finding was validated by Western blot (Fig. 7F).

To further confirm that AKT and CREB play a role in regulating PGC-1α expression, Western blot were performed on ARK1 cells treated with either AKT inhibitor Akti-1/2 or CBP/CREB inhibitor. The results showed markedly lower phosphorylated AKT (pS473) and CREB (pS133) levels as well as PGC-1α levels after treatment of Akti-1/2 or CBP/CREB inhibitor (Supplementary Fig. 4B). Western blot also showed that both HK1 and HK2 expression were reduced in a dose-dependent manner after treatment with Akti-1/2 but not CBP/CREB inhibitor (Supplementary Fig. 4C), suggesting that HK1/2 were specifically regulated by the CREB-independent AKT pathway. Furthermore, RYR1-overexpressing ARK1 cells were treated with Akti1-1/2 or CBP/CREB inhibitor and controls followed by Western blot for PGC-1α. The results showed that the effect of RYR1-enhanced PGC-1α expression was abrogated by Akti1-1/2 and CBP/CREB inhibitor (Fig. 7G), suggesting that RYR1 modulates PGC-1α expression through the AKT/CREB/PGC-1α pathway. To confirm that PGC-1α mediates the promoting effect of RYR1 on ATP production and mETC gene expression, PGC-1α-specific siRNAs or control siRNA was transfected into USC cells with or without stably overexpressing RYR1. The silencing efficiency of PGC-1α siRNAs in USC cells was validated by qPCR (Supplementary Fig. 4D). Cells transfected with full-length RYR1 and control siRNA had significantly higher levels of ATP production, which was reversed by treatment with PGC-1α siRNAs (Fig. 7H). In addition, the levels of mETC genes were also significantly higher in the RYR1-overexpressing cells compared to the mock transfectants. The enhanced expression of mETC genes in RYR1-overexpressing cells was abrogated by Akti1-1/2, CBP/CREB inhibitor, and PGC-1α siRNA in ARK1 cells (Fig. 7I). These results established the causal relationship of the RYR1-dependent AKT/CREB/PGC-1α pathway with elevated ATP level and mETC gene upregulation. In addition, the enhanced cell proliferation rate induced by RYR1 overexpression in ARK1 cells was abolished by inhibitor treatment (Fig. 7J).

To further confirm that RYR1-dependent regulation of the AKT/CREB/PGC-1α pathway is dependent on Ca^2+^ signals modulated by RYR1, [Ca^2+^]_i_ was buffered by incubating ARK1 cells with the cell permeant Ca^2+^ chelator BAPTA-AM (10 μM) for 1, 12, and 24 hours. The levels of p-CREB (S133) and p-AKT (S473) were significantly lowered in cells incubated with BAPTA-AM, accompanied with a significant reduction in the level of PGC-1α. The decrease in p-CREB (S133), p-AKT (S473), and PGC-1α protein levels after BAPTA treatment was attenuated in RYR1 knockdown ARK1 cells (Fig. 7K and Supplementary Fig. 4E). Additionally, the enhanced phosphorylation of AKT and CREB and overexpression of PGC-1α were again effectively obliterated by the Ca^2+^ chelators (Supplementary Fig. 4F). The mRNA level of PGC-1α induced by RYR1 overexpression was also attenuated by BAPTA treatment (Supplementary Fig. 4G). These results provide further evidence that RYR1-dependent Ca^2+^ signals mediate the enhanced AKT and CREB activities, leading to the upregulation of PGC-1α (Fig. 7L).

## Discussion

In this study, we sought to identify disease stage specific unfavorable prognostic biomarkers of USC that can be targeted by FDA-approved compounds and to investigate their underlying mechanisms for tumor growth. The major findings are, first, a meta-analysis of patient databases found that RYR1 is upregulated in USC tumors and its upregulation is associated with advanced disease stages in USC patients. Second, RYR1 is ubiquitously upregulated in USC cell lines; inhibition of RYR1 suppressed proliferation, motility, and enhanced apoptosis. Third, RYR1 upregulation is associated with increased resting [Ca^2+^]_i_, enhanced SR Ca^2+^ release and effective ER-mitochondrial Ca^2+^ transfer. Fourth, depletion or inhibition of RYR1 in USC cell lines revealed important RYR1-dependent gene regulation of mitochondrial ETC and glycolysis and casually related to alterations in bioenergetics properties. Fifth, the aberrant mitochondrial and glycolytic activities in the USC cells are mediated by RYR1 via the Ca^2+^-dependent AKT/CREB/PGC-1α and AKT/HK1/2 pathways. These results for the first time revealed the important roles of RYR1 in USC tumor progression, elucidated the RYR1-dependent pathological mechanisms, and suggested RYR1 as a potential target for the treatment of USC. Hence, repurposing the FDA-approved RYR1 blocker dantrolene can be an attractive additional therapy for USC.

RYR dysfunction has been linked to several detrimental diseases [29], including malignant hyperthermia [30] and catecholaminergic polymorphic ventricular tachycardia [31]. Functional RYRs has been reported in T-lymphoma cells [32], prostate cancer cells [33], neuroblastoma [34], and breast cancer cells [35, 36]. However, there is no related report on the role of RYRs, particularly RYR1, in the pathogenesis of USC. In this study, we found that RYR1 is the only RYR subtype upregulated in USC and their cell lines. Its upregulation is associated with altered Ca^2+^ homeostasis. The elevated [Ca^2+^]_i_ is apparently related to the increased Ca^2+^ release from RYR1, and probably RYR-dependent Ca^2+^ influx via the store-operated Ca^2+^ entry mechanism [37]. Increasing evidence suggests important roles of Ca^2+^ signaling in cancer pathogenesis [38]. RYR1 may represent a major aberrant Ca^2+^ mechanism in USC because its expression and activity are strongly linked to the malignant phenotypes of enhanced proliferation, migration, and apoptosis resistance.

RYR1 can exert its mitogenic influence through multiple signaling mechanisms. It provides diverse Ca^2+^ signals for the activation of Ca^2+^-sensitive pathways to trigger tumorigenic gene expression. Our data showed that the expression level of mETC enzymes were strongly dependent on the availability of RYR1. The reduction of OXPHOS, oxygen consumption, ATP production, mitochondrial membrane depolarization and increase in ROS level in RYR1-depleted or dantrolene-treated USC cells were also observed. Altered mitochondrial bioenergetics and biosynthetic states have long been implicated in tumorigenesis [39]. In USC, there are frequent mutations in somatic D-loop mitochondrial DNA, which contains essential transcription and replication elements; mutations in this region might alter the mitochondrial functions [40]. Hence, our results provide the first evidence that RYR1 is responsible, at least in part, for the regulation of mitochondrial mETC activity and metabolism in USC.

Analysis of signaling pathways showed that RYR1 regulates mETC enzyme expression through the AKT/CREB/PGC-1α pathway. PI3K/AKT activation is well recognized as an important signaling pathway, regulating cell growth, motility, survival, and metabolism in USC cells [41, 42]. Studies showed that PI3K/AKT is persistently activated in USC tissues [43], and its hyperactivation is thought to be a significant contributor to chemotherapy resistance in ovarian cancer [44]. RYR1-dependent increase in [Ca^2+^]_i_ can activate the PI3K/AKT pathway by Ca^2+^/calmodulin binding to the SH3 domain of PI3K p85 subunit [45], leading to the conversion of PIP2 to PIP3, which binds to both PDK1 and AKT to facilitate phosphorylation of T308 in the “activation loop”, and subsequent phosphorylation at S473 to stimulate full AKT activity. The activated AKT phosphorylates CREB at S133 [46] and stimulates PGC-1α expression via a CREB-dependent mechanism. Moreover, CREB can be activated synergistically by the increased [Ca^2+^]_i_ through CaMKIV [47]. This RYR1-dependent pathway was effectively disrupted by the suppression of RYR1 expression and activity; chelation of intracellular Ca^2+^ with the fast Ca^2+^ buffer BAPTA; inhibition of AKT or CREB with Akti-1/2 or CBP/CREB inhibitor, respectively. Thus, our results provide direct evidence for each major step in the RYR1-mediated activation of the AKT/CREB/ PGC-1α signaling cascade and its downstream regulation of mETC expression.

In addition to the transcriptional regulation of mETC enzymes, RYR1-dependent Ca^2+^ signals can modulate mitochondrial metabolism through “ER-mitochondrial Ca^2+^ transfer” [48]. Ca^2+^ released from SR/ER enters mitochondria through voltage-dependent anion channels in the outer membrane and mitochondrial Ca^2+^ uniporters in the inner mitochondrial membrane [49, 50]. The Ca^2+^-dependent stimulation of OXPHOS provide the H^+^ gradient to maintain the mitochondrial membrane potential for ATP synthesis by F_1_/F_0_ ATPase, and inhibition of RYR1 in the cancer cells led to mitochondrial membrane depolarization and reduction in ATP production [51]. Moreover, suppression of RYR1 activity led to dramatic increase in ROS production, presumably through induction of mitochondrial permeability transition pore opening [52], and apoptosis. Several studies have demonstrated that elevated [Ca^2+^]_m_ plays important roles in cancer progression by promoting proliferation, cell migration and conferring apoptosis resistance [53–55]. This study demonstrated the RYR-mediated ER-mitochondrial Ca^2+^ transfer in USC cells by simultaneous recording of ER and mitochondrial Ca^2+^; and established its acute influences on OXPHOS, ATP production, and ROS production by inhibiting RYR1.

It is noteworthy that RYR1-dependent AKT activation contributes to enhanced aerobic glycolysis. This is evidenced by the fact that RYR1 shRNA and dantrolene reduced glycolytic intermediate metabolites and lactic acid production. Moreover, RYR1 knockdown and inhibition of AKT with Akti-1/2 significantly reduced the expression of the hexokinases HK1 and HK2. This is consistent with the well-documented role of AKT in the regulation of mitochondrial hexokinases [56] and the reports that enhanced HK2 activity is an important mechanism for tumor survival [57].

The abovementioned findings in USC cell lines are corroborated by our observations of USC in xenograft mouse models in vivo. RYR1 suppression in the USC xenograft mouse models dramatically reduced tumor mass and prolonged survival time. RYR1 suppression can be applied therapeutically for the treatment USC, which is supported by the test-of-concept experiment showing that the RYR1 antagonist dantrolene could effectively reduce USC tumor burden and progression in the xenograft mouse models.

Our study demonstrates the first time the feasibility of repurposing dantrolene in cancer treatment. In this study, dantrolene was administered by intraperitoneal injection of 5 mg/kg every 2 days, which is equivalent to 0.4 mg/kg for an average person of 70 kg, taking into account the surface/mass ratio [58]. Hence, the effective dose of dantrolene for USC treatment could be considerably lower than that used for acute malignant hyperthermia. The low effective dose of dantrolene has the benefit of reducing the possible side effects of muscle weakness, dizziness, diarrhea, nausea, and diarrhea that may occur in patients [14]. In fact, we observed no abnormalities in other organs, especially skeletal muscle and heart, in our dantrolene-treated mice.

There are several limitations remain to be solved. First, the mechanism by which RYR1 is upregulated in USC is unknown. Previous studies have identified a number of transcriptional factors for RYR1 expression in skeletal muscles [59]. Whether these factors play a role in regulating RYR1 expression in non-excitable USC cells remains to be determined. Second, chemotherapy resistance is considered a major contributor to mortality in USC patients [60]. The possible roles of RYR1 in chemoresistance, and the potential synergistic effects of dantrolene and common first-line therapeutic agents for the treatment of USC require future investigations.

## Conclusions

This study has discovered the important tumorigenic roles of RYR1 in USC progression, delineated the molecular mechanism by which RYR1-mediated Ca^2+^ signaling and mitochondrial reprogramming modulate the malignant phenotype of USC, and evaluated the potential of repurposing dantrolene in the treatment of USC. Further studies on determining the efficacy of using dantrolene alone or in combination with common first-line therapeutic agents will allow the development of new therapeutic strategies in USC treatment and the improvement of patient survival rates.

## Supplementary Information

**Additional file 1.**

**Additional file 2.**

## Data Availability

The dataset used or analyzed during the current study are available from the corresponding authors on reasonable request.

## Abbreviations

ATCC: American type culture collection
ATP: Adenosine triphosphate
CRE: cAMP response element
CREB: cAMP response element-binding protein
NAD: Nicotinamide adenine dinucleotide
ECAR: Extracellular acidification rate
EEC: Endometrioid endometrial cancer
ER: Endoplasmic reticulum
FBS: Fetal bovine serum
FDA: Food and Drug Administration
G6P: Glucose-6-phosphate
GEO: Gene expression omnibus
HGSC: High-grade serous ovarian cancer
mETC: Mitochondrial electron transport chain
RIPA: Radioimmunoprecipitation assay
ROS: Reactive oxygen species
RPPA: Reverse phase protein array
OCR: Oxygen consumption rate
OXPHOS: Oxidative phosphorylation
RTCA: Real-time cell analysis
RYR1: Ryanodine receptor 1
SAM: Significance Analysis of Microarray
TCA: Tricarboxylic acid cycle
TCGA: The Cancer Genome Atlas
