# Supplementary material for: Ryanodine receptor 1-mediated Ca2+ signaling and mitochondrial reprogramming modulate uterine serous cancer malignant phenotypes
Source: J Exp Clin Cancer Res. 2022 Aug 11;41:242. doi: 10.1186/s13046-022-02419-w (PMC9373370; doi:10.1186/s13046-022-02419-w)
Supplement: Supplementary file 1 — Additional file 1. [file 13046_2022_2419_MOESM1_ESM.docx]

**Supplementary Experimental Procedures**

**Immunohistochemical (IHC) analysis**

Formalin-fixed, paraffin-embedded tumors from mice and patients were sectioned. The slides were deparaffinized, and antigen retrieval was conducted in pH 6.0 citrate buffer (Sigma), followed by hematoxylin and eosin staining and immunolocalization of Ki-67 (Invitrogen), Cleaved Caspase 3 (Cell Signaling), CD31 (Cell Signaling), and PGC-1α (Cell Signaling). Target protein expression was visualized using a betazoid 3,30-diaminobenzidine chromogen kit (Biocare Medical). Images of stained tumor sections were taken by an Olympus microscope. Five images per experimental group were scored to determine positive cells.

**Reverse transcription (RT)-quantitative PCR**

Total RNA was extracted from control or treated groups of cells or mice using TRIzol reagent (Thermo Fisher Scientific) following the manufacturer’s instructions. The NanoDrop 2000 (Thermo Fisher Scientific) was used to determine the concentration and integrity of total RNA. cDNA was synthesized via ImProm-II Reverse Transcription System kit (Promega). Amplification of indicated genes was conducted using SsoAdvanced Universal SYBR Green Supermix (Bio-Rad). Gene-specific primers were synthesized from Sigma (Table S3). Quantitative PCR analysis was executed using CFX Connect Touch Real-Time PCR Detection System (Bio-Rad).

**Targeted metabolite measurement**

Glycolytic and TCA metabolites in cells were analyzed by targeted metabolites analysis using liquid chromatograph/Mass Spectrometry (LC/MS) as described by Kettner et al. [61].

*Internal standards and quality control*

Mouse pooled liver or pooled serum samples were run together with the experimental samples to test the reproducibility of the whole process. Internal standards, including injection standards, process standards, and alignment standards, were applied to confirm QA/QC targets to control for experimental variability. The reproducibility of the profiling process was solved by examining instrument variation alone and the overall process. Matrix-free internal standards were used for monitoring instrument variation and analyzed by MRM. The median coefficient of variation value for internal standards was 5%, < 5% for technical replicates of each experimental sample.

*Separation of metabolites*

Normal phase chromatography was applied to extract metabolites from cells or mouse tissues using solvents containing water (solvent A) with ammonium acetate (pH 9.9) and acetonitrile (ACN, solvent B). The binary pump flow rate was 0.2 mL/min with a gradient spanning 80% B to 2% B over 20 min, followed by 2% B to 80% B for 5 min and 80% B for 13 min. During the separation period, the flow rate was gradually changed from 0.2 mL/min (0-20 min) to 0.3 mL/min (20-25 min), and then 0.35 mL/min (25-30 min), 0.4 mL/min (30-37.99 min), and finally to 0.2 mL/min (5 min). Luna Amino columns (Phenominex) were used to separate metabolites for LC/MS analyses.

*Liquid chromatography/mass spectrometry*

Metabolites were examined using 1290 SL Rapid resolution LC and a 6490 triple quadrupole mass spectrometer (Agilent Technologies) with unbiased profiling platform. A dual electrospray ionization source was used to examine experimental samples in both positive and negative ionization modes. Data collection was controlled by the Mass Hunter workstation data acquisition software.

**Table S1. List of significantly upregulated genes that are correlated with either advanced disease stage or overall survival in USC patients**

| **Gene** | **Fold change in USC vs EEC** | **Fold change in USC vs Normal** | **Hazard ratio (Disease stage)** | **p-value (Disease stage)** | **Hazard ratio (Overall survival)** | **p-value (Overall survival)** | **FDA-approved drug(s)** |
| --- | --- | --- | --- | --- | --- | --- | --- |
| GPR158 | 20.219 | 28.594 | 4.130 | 0.009 | 0.997 | n.s. | N/A |
| IGF2BP1 | 20.208 | 131.214 | 4.108 | 0.009 | 1.000 | n.s. | N/A |
| NKAIN4 | 14.685 | 18.827 | 4.158 | 0.008 | 0.999 | n.s. | N/A |
| VGLL1 | 13.235 | 64.903 | 4.441 | 0.008 | 1.001 | n.s. | N/A |
| TMEM63C | 11.773 | 7.094 | 4.583 | 0.005 | 1.003 | n.s. | N/A |
| PRSS50 | 11.095 | 11.211 | 4.197 | 0.009 | 1.000 | n.s. | N/A |
| FIGNL2 | 10.598 | 3.151 | 5.111 | 0.003 | 0.997 | n.s. | N/A |
| CDH6 | 10.565 | 7.323 | 2.528 | n.s. | 1.000 | 0.008 | N/A |
| RYR1 | 9.418 | 6.379 | 4.302 | 0.008 | 0.999 | n.s. | Dantrolene, Caffeine, Tetracaine, Calcium citrate, Calcium Phosphate, Calcium Phosphate dihydrate |
| BMP7 | 8.003 | 7.113 | 5.578 | 0.002 | 1.000 | n.s. | N/A |
| DUSP9 | 6.447 | 24.962 | 5.741 | 0.002 | 0.992 | n.s. | N/A |
| SCN4A | 6.389 | 3.232 | 4.232 | 0.007 | 0.994 | n.s. | Zonisamide, Propofol, Parachlorophenol, Lidocaine, Diclofenac |
| DLL3 | 6.272 | 17.304 | 4.226 | 0.006 | 0.993 | n.s. | N/A |
| ANKLE1 | 6.203 | 10.240 | 4.395 | 0.006 | 0.998 | n.s. | N/A |
| CBS | 5.227 | 6.417 | 4.023 | 0.007 | 1.000 | n.s. | Pyridoxal phosphate, Cysteine, Ademetionine |
| CLCNKB | 5.031 | 3.286 | 6.100 | 0.002 | 0.991 | n.s. | N/A |
| EMID2 | 4.525 | 37.307 | 4.823 | 0.006 | 0.999 | n.s. | N/A |
| HMGA2 | 4.324 | 11.638 | 5.599 | 0.003 | 1.000 | n.s. | N/A |
| GDPD2 | 3.890 | 5.807 | 4.356 | 0.006 | 1.006 | n.s. | N/A |
| KIRREL2 | 3.749 | 4.164 | 5.077 | 0.003 | 0.992 | n.s. | N/A |
| NPW | 3.705 | 4.064 | 4.586 | 0.004 | 0.997 | n.s. | N/A |
| DLX3 | 3.665 | 18.168 | 4.364 | 0.007 | 0.998 | n.s. | N/A |
| GAL | 3.416 | 27.045 | 3.223 | n.s. | 1.004 | 0.007 | N/A |
| UPK3B | 3.391 | 14.724 | 4.034 | 0.009 | 0.999 | n.s. | N/A |
| SPSB4 | 3.160 | 2.683 | 5.044 | 0.005 | 0.998 | n.s. | N/A |
| NR6A1 | 3.152 | 4.881 | 4.259 | 0.008 | 1.031 | n.s. | N/A |
| TCF15 | 3.134 | 2.083 | 4.178 | 0.007 | 0.995 | n.s. | N/A |
| COL11A2 | 2.944 | 4.611 | 4.471 | 0.005 | 0.999 | n.s. | N/A |
| INSL3 | 2.760 | 5.871 | 4.110 | 0.008 | 1.016 | n.s. | N/A |
| PLEKHG4 | 2.717 | 2.443 | 4.474 | 0.005 | 0.999 | n.s. | N/A |
| SH2D5 | 2.715 | 2.245 | 4.071 | 0.007 | 0.992 | n.s. | N/A |
| KCNQ3 | 2.572 | 4.572 | 3.563 | n.s. | 1.022 | 0.001 | Ezogabine, Meclofenamic acid, Amitriptyline, Diclofenac, Gabapentin |
| LY6K | 2.489 | 9.623 | 4.356 | 0.008 | 1.001 | n.s. | N/A |
| CCBP2 | 2.437 | 3.897 | 4.678 | 0.006 | 1.001 | n.s. | N/A |
| MMP7 | 2.428 | 4.685 | 4.426 | 0.006 | 1.000 | n.s. | N/A |
| PSAT1 | 2.392 | 11.296 | 4.815 | 0.005 | 1.000 | n.s. | Pyridoxal phosphate, Glutamic acid |
| ARL4C | 2.350 | 4.721 | 3.787 | n.s. | 1.000 | 0.009 | N/A |
| DLX4 | 2.347 | 18.134 | 6.152 | 0.002 | 0.993 | n.s. | N/A |
| CD47 | 2.247 | 2.321 | 4.698 | 0.004 | 1.000 | n.s. | N/A |
| EPPK1 | 2.235 | 5.913 | 4.456 | 0.006 | 1.000 | 0.001 | N/A |
| KAZ | 2.200 | 2.822 | 4.359 | 0.006 | 0.999 | n.s. | N/A |
| HSF2BP | 2.178 | 2.049 | 5.371 | 0.004 | 0.983 | n.s. | N/A |
| MACC1 | 2.167 | 4.271 | 4.158 | 0.007 | 1.001 | n.s. | N/A |
| STK32C | 2.126 | 2.094 | 4.107 | 0.008 | 0.999 | n.s. | N/A |
| PARD6B | 2.109 | 3.216 | 4.042 | 0.008 | 0.999 | n.s. | N/A |
| SLC16A5 | 2.095 | 2.052 | 4.078 | 0.008 | 1.000 | n.s. | Pyruvic acid |
| C1QL4 | 2.064 | 12.775 | 4.061 | 0.008 | 0.999 | n.s. | N/A |
| SLC4A11 | 2.046 | 16.332 | 4.065 | 0.009 | 1.000 | n.s. | N/A |
| CTCFL | 2.020 | 13.276 | 4.430 | 0.009 | 1.000 | n.s. | N/A |
| C19orf57 | 2.016 | 3.021 | 5.569 | 0.002 | 0.994 | n.s. | N/A |
| DCDC2 | 2.007 | 2.630 | 4.158 | 0.007 | 1.000 | n.s. | N/A |
| n.s.: not significant; N/A: not applicable | | | |  |  |  |  |

**Table S2. List of genes in NDUF, SDH, COX and mitochondrial ATPase subfamilies that show in GEO dataset GSE24537**

| **Gene Name** | **R^2^** | **P -value** |
| --- | --- | --- |
| NDUFB1 | 0.01412 | 0.744 |
| NDUFB2 | 0.1441 | 0.279 |
| NDUFB5 | 0.1441 | 0.279 |
| NDUFB6 | 0.4695 | 0.029 |
| NDUFB8 | 0.04802 | 0.543 |
| NDUFB11 | 0.1520 | 0.265 |
| SDHB | 0.4562 | 0.032 |
| SDHC | 0.4388 | 0.037 |
| COX5 | 0.046 | 0.046 |
| COX6A1 | 0.098 | 0.098 |
| COX6C | 0.098 | 0.098 |
| COX7B | 0.084 | 0.084 |
| COX8A | 0.07336 | 0.449 |
| COX8A2 | 0.5708 | 0.012 |
| COX8C | 0.04702 | 0.037 |
| ATP5C1 | 0.052 | 0.526 |
| ATP5F1 | 0.1015 | 0.37 |
| ATP5G3 | 0.1194 | 0.328 |
| ATP5H | 0.08843 | 0.404 |
| ATP5I | 0.6253 | 0.006 |
| ATP5J | 0.1848 | 0.215 |
| ATP5L | 0.1564 | 0.258 |
| ATP5S | 0.023518 | 0.687 |

**Table S3. List of reagents and chemicals used**

| **Reagents and chemicals** | **Company** | **Catalog no.** |
| --- | --- | --- |
| **Antibodies** |  |  |
| Anti-RYR1 | Novus Biologicals | 46140002 |
| Anti-Ki-67 | Cell Signaling | 12202 |
| Anti-Cleaved Casplase-3 | Cell Signaling | 9661 |
| Anti-CD31 | Cell Signaling | 77699 |
| Anti-NDUBF4 | Abcam | ab110243 |
| Anti-SDHA | ABclonal | A2594 |
| Anti-COXIV | Proteintech | 11242-1AP |
| Anti-ATP5a | ABclonal | A5884 |
| Anti-β-actin | Sigma | A5441 |
| Anti-hexokinase II | Cell Signaling | 2867 |
| Anti-PGC-1α | Cell Signaling | 2178 |
| Anti-phosphorylated AKT(T308) | Cell Signaling | 13038 |
| Anti-phosphorylated AKT(S473) | Cell Signaling | 4060 |
| Anti-AKT (pan) | Cell Signaling | 4691 |
| Anti-phosphorylated CREB (S133) | Cell Signaling | 9198 |
| Anti-CREB | Cell Signaling | 4820 |
| Peroxidase-conjugated secondary antibody (Mouse) | Abcam | ab205719 |
| Peroxidase-conjugated secondary antibody (Rabbit) | Abcam | ab205718 |
| **Viral particles** |  |  |
| RYR1 Mission shRNA lentiviral transduction particles-1 | Sigma | TRCN0000174210 |
| RYR1 Mission shRNA lentiviral transduction particles-2 | Sigma | TRCN0000359103 |
| pLKO.1-puro non-target shRNA control transduction particles | Sigma | SHC016V |
| CRISPR/dCas9 human RYR1 lentiviral activation particles | Santa Cruz Biotechnology | sc-401470-LAC |
| Control lentiviral activation particles | Santa Cruz Biotechnology | sc-437282 |
| **Oligonucleotides and probes** |  |  |
| siRNA Universa Negative Control #1 | Sigma | SIC001 |
| PPARGC1A-siRNA | Sigma | NM_13261_00063323 |
| PPARGC1A-siRNA | Sigma | NM_13261_00063324 |
| hSDHA-F: TGGGAACAAGAGGGCATCTG | Thermo Fisher Scientific | N/A |
| hSDHA-R: CCACCACTGCATCAAATTCATG | Thermo Fisher Scientific | N/A |
| hNDUBF4-F:GCCATAAGAGCCCTTGTTC | Thermo Fisher Scientific | N/A |
| hNDUBF4-R:ACGAAGCAAGGCAGGATTTTC | Thermo Fisher Scientific | N/A |
| hATP5a-F:GCCCTTACTCTGCCTGTTCCA | Thermo Fisher Scientific | N/A |
| hATP5a-R:GCGGAGGCAACAGAGACATCTGA | Thermo Fisher Scientific | N/A |
| hCOXIV-F:TCGGTTTCACCGCGCGCTCGTTAT | Thermo Fisher Scientific | N/A |
| hCOXIV-R:TGTCCAGCATCCTCTTGGTCTG | Thermo Fisher Scientific | N/A |
| hBeta-actin-F: GCTGCGTGTGGCTCCCGAGGAG | Thermo Fisher Scientific | N/A |
| hBeta-actin-R: ATCTTCATTGTGCTGGGTGCCAG | Thermo Fisher Scientific | N/A |
| RYR1 probe | Thermo Fisher Scientific | Hs00166991_m1 |
| human HPRT1 endogenous control probe | Thermo Fisher Scientific | 4333768F |
| **Commercially available assay kits** |  |  |
| BCA Protein Assay kit | Roche | 1836170 |
| Mitochondrial ROS Detection assay kit | Cayman | 701600 |
| FITC Annexin V apoptosis Detection Kit I | BD Pharmingen | 556547 |
| Reactive oxygen species (ROS) detection assay kit | Biovision | K936-100 |
| CellTiter 2.0 assay | Promega | G9241 |
| NAD/NADH-Glo and NADP/NADPH-Glo assays | Promega | G9071 |
| Cytochrome c oxidase assay kit | Sigma | CYTOCOX1 |
| ATP colorimetric/fluorometric assay kit | Biovision | K354-100 |
| Deproteinization sample preparation kit | Biovision | K808-200 |
| Mitochondria isolation kit | Invitrogen | 89874 |
| MitoProbe JC-1 assay kit | Thermo Fisher Scientific | M34152 |
| Seahorse XF cell mito stress kit | Agilent Technologies | 103015-100 |
| Seahorse XF real-time ATP rate assay kit | Agilent Technologies | 103592-100 |
| **Chemicals and reagents** |  |  |
| Dantrolene | Sigma | 1163140 |
| AKT inhibitor | Tocris | 5773 |
| CBP-CREB interaction inhibitor | Sigma | 217505 |
| Puromycin | GIBCO | A1113803 |
| Protamine sulfate | Sigma | P4020 |
| G418 (Neomycin) | CORNING | 30-234-CI |
| Hygromycine B solution | Santa Cruz Biotechnology | 31282-04-9 |
| Lipofectamine 2000 | Invitrogen | 11668019 |
| Trizol Reagent | Invitrogen | 1610798 |
| SsoAdvanced Universal SYBR Green Supermix | Bio-Rad | 1725270 |
| Improm-II reverse transcription system | Promega | A3800 |
| ddPCR supmix | Bio-Rad | 186-3024 |
| EDTA-free protease inhibitor cocktail tablets | Roche | 11873580001 |
| RIPA buffer | Thermo Fisher Scientific | 89901 |
| 1.5 M Tris-HCL, pH8.8 | Bio-Rad | 1610798 |
| 0.5M Tris-HCL, pH6.8 | Bio-Rad | 1610799 |
| Laemmli Sample buffer (2x) | Bio-Rad | 1610737 |
| Nitrocellulose transfer membrane | Bio-Rad | 1620115 |
| PageRuler plus prestained protein ladder | Thermo Fisher Scientific | 26619 |
| HyClone RPMI-1640 media | Fisher Scientific | SH3002701 |
| Heat inactivated serum | Sigma | F4135 |
| Trypsin EDTA | Fisher Scientific | MT25053CI |
| PEN/STREP | Fisher Scientific | MT30002CI |
| Seahorse XF RPMI medium | Agilent Technologies | 103576-100 |
| Seahorse XFp calibrant solution | Agilent Technologies | 100840-000 |
