# Supplementary material for: Ryanodine receptor 1-mediated Ca2+ signaling and mitochondrial reprogramming modulate uterine serous cancer malignant phenotypes
Source: J Exp Clin Cancer Res. 2022 Aug 11;41:242. doi: 10.1186/s13046-022-02419-w (PMC9373370; doi:10.1186/s13046-022-02419-w)
Supplement: Supplementary file 2 — Additional file 2. [file 13046_2022_2419_MOESM2_ESM.pptx]

## Slide 1
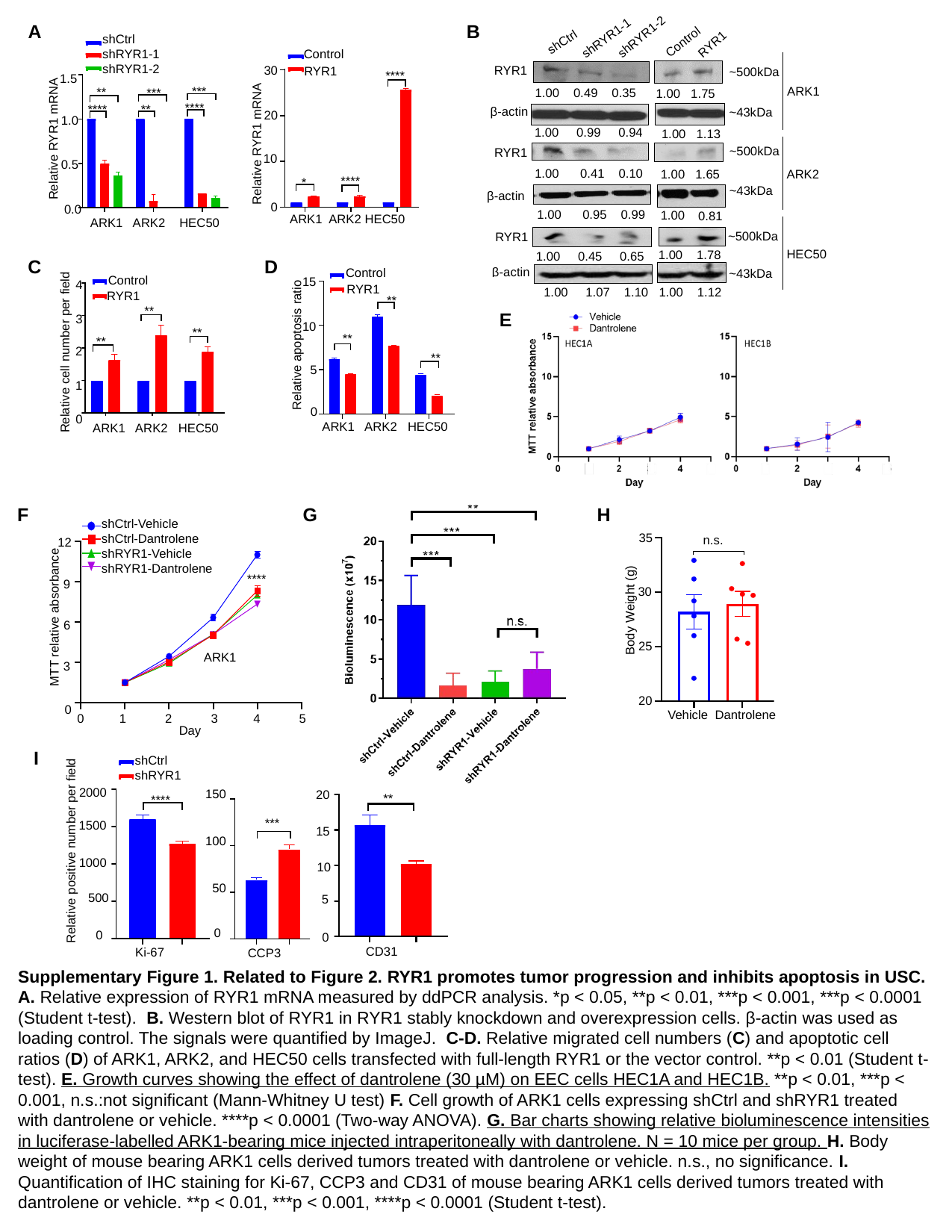

A
B
shCtrl
shRYR1-1
shRYR1-2
***
**
**
***
****
*
**
****
1.5
1.0
0.5
0.0
ARK1 ARK2 HEC50
Relative RYR1 mRNA
Control
RYR1
30
20
10
0
ARK1 ARK2 HEC50
****
***
****
*
Relative RYR1 mRNA
shRYR1-2
shRYR1-1
shCtrl
Control
RYR1
RYR1
~500kDa
ARK1
1.00 0.49 0.35
1.00 1.75
β-actin
~43kDa
1.00 0.99 0.94
1.00 1.13
~500kDa
RYR1
1.00 0.41 0.10
1.00 1.65
ARK2
~43kDa
β-actin
1.00 0.95 0.99
1.00
 0.81
~500kDa
RYR1
HEC50
1.00 1.78
1.00 0.45 0.65
β-actin
~43kDa
1.00 1.07 1.10
1.00 1.12
C
D
Control
RYR1
4
3
2
1
0
**
**
**
**
Relative cell number per field
ARK1 ARK2 HEC50
Control
RYR1
15
10
5
0
**
**
*
**
Relative apoptosis ratio
ARK1 ARK2 HEC50
E
F
G
H
shCtrl-Vehicle
shCtrl-Dantrolene
shRYR1-Vehicle
shRYR1-Dantrolene
12
6
3
0
9
****
MTT relative absorbance
ARK1
0 1 2 3 4 5
Day
35
30
25
20
Vehicle Dantrolene
n.s.
Body Weight (g)
2000
1500
Relative positive number per field
1000
500
0
shCtrl
shRYR1
150
100
50
0
CCP3
20
15
10
5
0
CD31
****
**
***
Ki-67
I
Supplementary Figure 1. Related to Figure 2. RYR1 promotes tumor progression and inhibits apoptosis in USC.
A. Relative expression of RYR1 mRNA measured by ddPCR analysis. *p < 0.05, **p < 0.01, ***p < 0.001, ***p < 0.0001 (Student t-test). B. Western blot of RYR1 in RYR1 stably knockdown and overexpression cells. β-actin was used as loading control. The signals were quantified by ImageJ. C-D. Relative migrated cell numbers (C) and apoptotic cell ratios (D) of ARK1, ARK2, and HEC50 cells transfected with full-length RYR1 or the vector control. **p < 0.01 (Student t-test). E. Growth curves showing the effect of dantrolene (30 µM) on EEC cells HEC1A and HEC1B. **p < 0.01, ***p < 0.001, n.s.:not significant (Mann-Whitney U test) F. Cell growth of ARK1 cells expressing shCtrl and shRYR1 treated with dantrolene or vehicle. ****p < 0.0001 (Two-way ANOVA). G. Bar charts showing relative bioluminescence intensities in luciferase-labelled ARK1-bearing mice injected intraperitoneally with dantrolene. N = 10 mice per group. H. Body weight of mouse bearing ARK1 cells derived tumors treated with dantrolene or vehicle. n.s., no significance. I. Quantification of IHC staining for Ki-67, CCP3 and CD31 of mouse bearing ARK1 cells derived tumors treated with dantrolene or vehicle. **p < 0.01, ***p < 0.001, ****p < 0.0001 (Student t-test).

## Slide 2
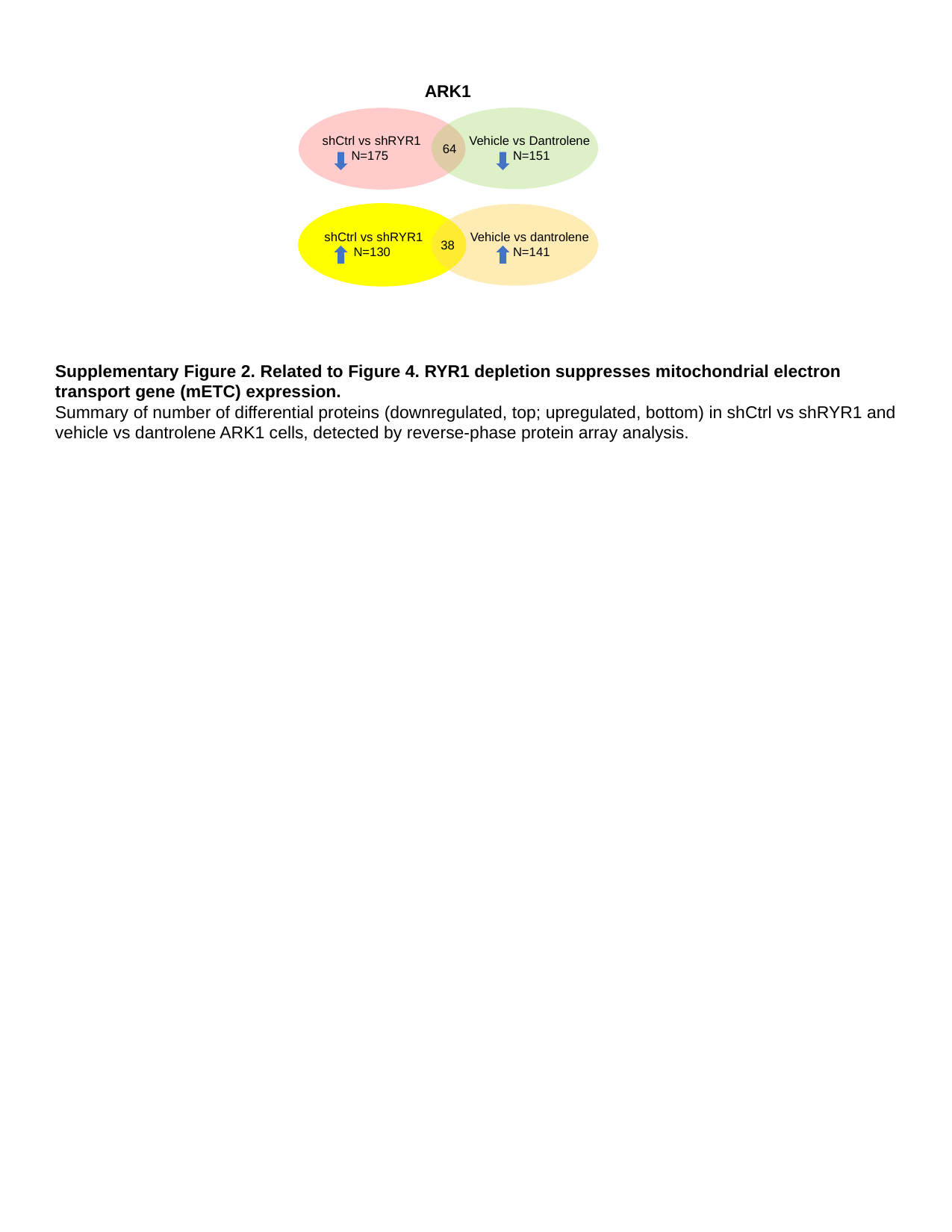

ARK1
 shCtrl vs shRYR1
N=175
Vehicle vs Dantrolene
N=151
64
 shCtrl vs shRYR1
N=130
Vehicle vs dantrolene
N=141
38
Supplementary Figure 2. Related to Figure 4. RYR1 depletion suppresses mitochondrial electron transport gene (mETC) expression.
Summary of number of differential proteins (downregulated, top; upregulated, bottom) in shCtrl vs shRYR1 and vehicle vs dantrolene ARK1 cells, detected by reverse-phase protein array analysis.

## Slide 3
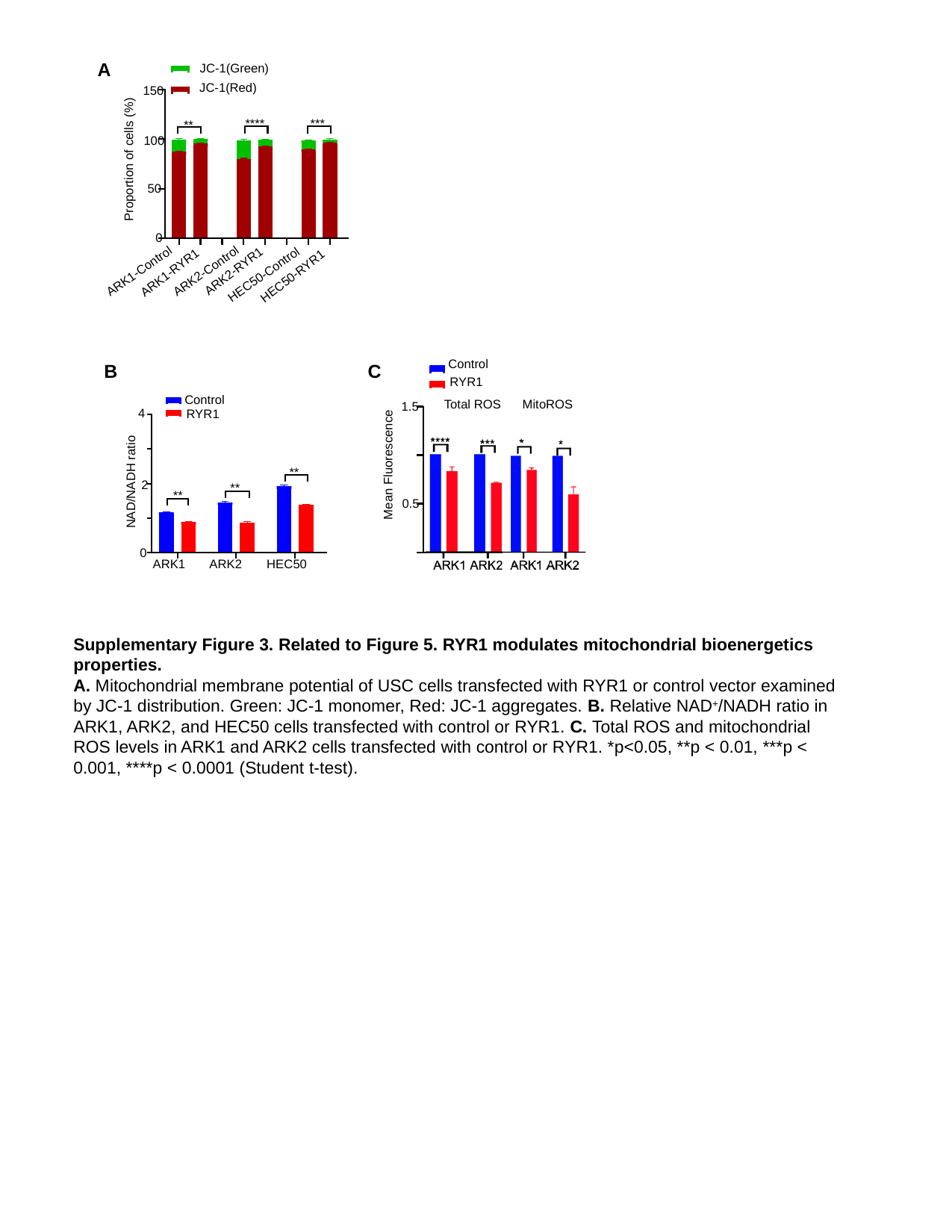

JC-1(Green)
JC-1(Red)
150
50
0
****
***
***
**
100
Proportion of cells (%)
ARK2-RYR1
ARK2-Control
ARK1-Control
ARK1-RYR1
HEC50-Control
HEC50-RYR1
A
Control
RYR1
*
**
****
*
*
***
Mean Fluorescence
 ARK1 ARK2
 ARK1 ARK2
MitoROS
Total ROS
1.5
0.5
B
C
Control
RYR1
4
2
0
ARK1 ARK2 HEC50 OVCA432
 NAD/NADH ratio
**
**
**
**
Supplementary Figure 3. Related to Figure 5. RYR1 modulates mitochondrial bioenergetics properties.
A. Mitochondrial membrane potential of USC cells transfected with RYR1 or control vector examined by JC-1 distribution. Green: JC-1 monomer, Red: JC-1 aggregates. B. Relative NAD+/NADH ratio in ARK1, ARK2, and HEC50 cells transfected with control or RYR1. C. Total ROS and mitochondrial ROS levels in ARK1 and ARK2 cells transfected with control or RYR1. *p<0.05, **p < 0.01, ***p < 0.001, ****p < 0.0001 (Student t-test).

## Slide 4
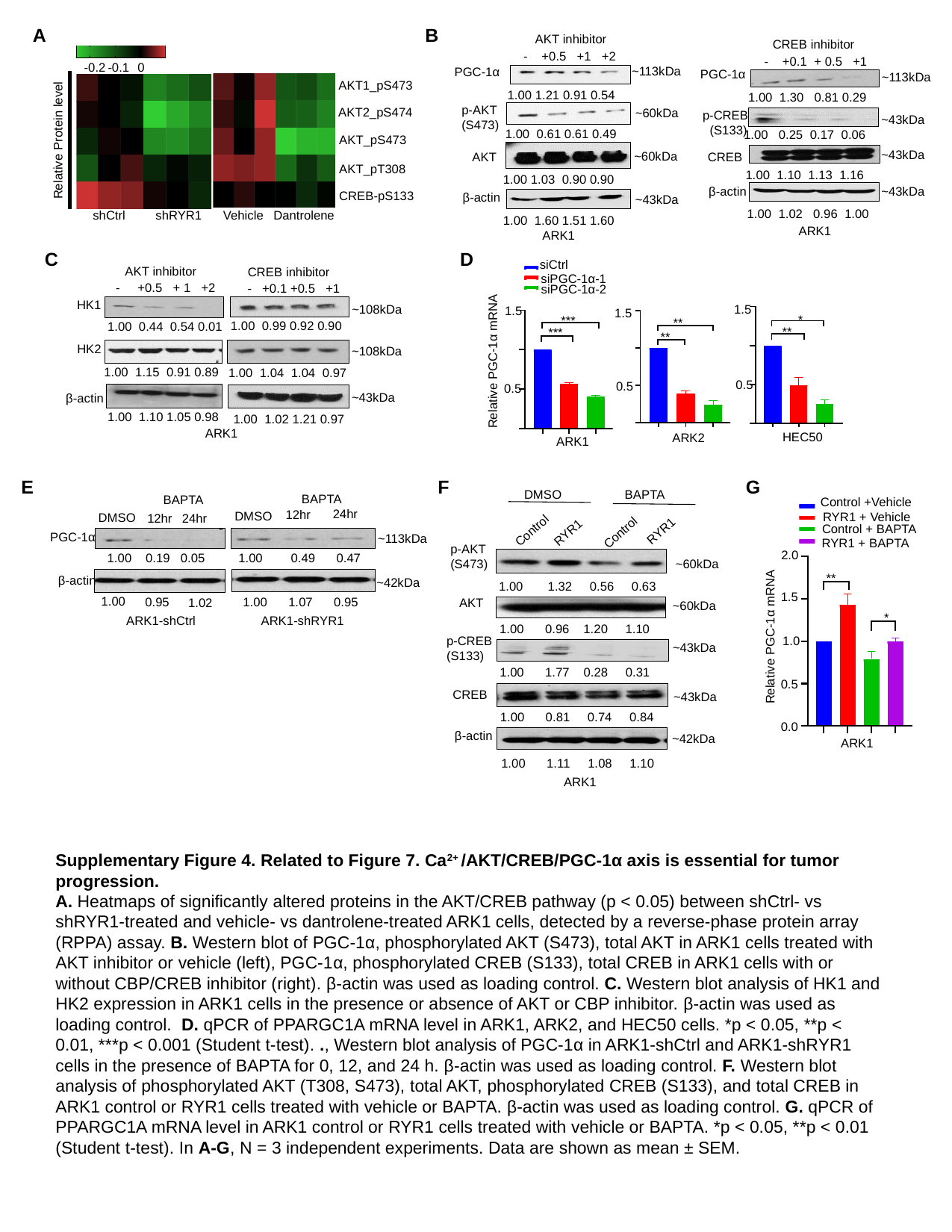

A
B
AKT inhibitor
- +0.5 +1 +2
~113kDa
PGC-1α
1.00 1.21 0.91 0.54
p-AKT
(S473)
~60kDa
1.00 0.61 0.61 0.49
~60kDa
AKT
1.00 1.03 0.90 0.90
β-actin
~43kDa
1.00 1.60 1.51 1.60
ARK1
CREB inhibitor
- +0.1 + 0.5 +1
PGC-1α
~113kDa
1.00 1.30 0.81 0.29
p-CREB
 (S133)
~43kDa
1.00 0.25 0.17 0.06
~43kDa
CREB
1.00 1.10 1.13 1.16
~43kDa
β-actin
1.00 1.02 0.96 1.00
ARK1
-0.2
-0.1
0
AKT1_pS473
AKT2_pS474
AKT_pS473
AKT_pT308
CREB-pS133
Vehicle
Dantrolene
Relative Protein level
shCtrl
shRYR1
C
D
siCtrl
siPGC-1α-1
siPGC-1α-2
1.5
0.5
HEC50
1.5
0.5
ARK1
1.5
0.5
ARK2
**
**
*
**
***
***
Relative PGC-1α mRNA
AKT inhibitor
CREB inhibitor
- +0.5 + 1 +2
HK1
1.00 0.44 0.54 0.01
- +0.1 +0.5 +1
~108kDa
1.00 0.99 0.92 0.90
HK2
~108kDa
1.00 1.15 0.91 0.89
1.00 1.04 1.04 0.97
~43kDa
β-actin
1.00 1.10 1.05 0.98
1.00 1.02 1.21 0.97
ARK1
E
F
G
BAPTA
DMSO
Control
RYR1
RYR1
Control
p-AKT
(S473)
~60kDa
 1.00 1.32 0.56 0.63
AKT
~60kDa
1.00 0.96 1.20 1.10
CREB
~43kDa
1.00 0.81 0.74 0.84
β-actin
~42kDa
1.00 1.11 1.08 1.10
ARK1
p-CREB
(S133)
~43kDa
1.00 1.77 0.28 0.31
BAPTA
24hr
12hr
DMSO
BAPTA
DMSO
24hr
12hr
PGC-1α
1.00 0.19 0.05
1.00 0.49 0.47
1.00
0.95
1.00 1.07 0.95
1.02
ARK1-shRYR1
ARK1-shCtrl
~113kDa
~42kDa
β-actin
Control +Vehicle
RYR1 + Vehicle
Control + BAPTA
RYR1 + BAPTA
2.0
1.5
1.0
0.5
0.0
Relative PGC-1α mRNA
**
*
ARK1
Supplementary Figure 4. Related to Figure 7. Ca2+ /AKT/CREB/PGC-1α axis is essential for tumor progression.
A. Heatmaps of significantly altered proteins in the AKT/CREB pathway (p < 0.05) between shCtrl- vs shRYR1-treated and vehicle- vs dantrolene-treated ARK1 cells, detected by a reverse-phase protein array (RPPA) assay. B. Western blot of PGC-1α, phosphorylated AKT (S473), total AKT in ARK1 cells treated with AKT inhibitor or vehicle (left), PGC-1α, phosphorylated CREB (S133), total CREB in ARK1 cells with or without CBP/CREB inhibitor (right). β-actin was used as loading control. C. Western blot analysis of HK1 and HK2 expression in ARK1 cells in the presence or absence of AKT or CBP inhibitor. β-actin was used as loading control. D. qPCR of PPARGC1A mRNA level in ARK1, ARK2, and HEC50 cells. *p < 0.05, **p < 0.01, ***p < 0.001 (Student t-test). ., Western blot analysis of PGC-1α in ARK1-shCtrl and ARK1-shRYR1 cells in the presence of BAPTA for 0, 12, and 24 h. β-actin was used as loading control. F. Western blot analysis of phosphorylated AKT (T308, S473), total AKT, phosphorylated CREB (S133), and total CREB in ARK1 control or RYR1 cells treated with vehicle or BAPTA. β-actin was used as loading control. G. qPCR of PPARGC1A mRNA level in ARK1 control or RYR1 cells treated with vehicle or BAPTA. *p < 0.05, **p < 0.01 (Student t-test). In A-G, N = 3 independent experiments. Data are shown as mean ± SEM.
